# Genomic dissection of conserved transcriptional regulation in intestinal epithelial cells

**DOI:** 10.1371/journal.pbio.2002054

**Published:** 2017-08-29

**Authors:** Colin R. Lickwar, J. Gray Camp, Matthew Weiser, Jordan L. Cocchiaro, David M. Kingsley, Terrence S. Furey, Shehzad Z. Sheikh, John F. Rawls

**Affiliations:** 1 Department of Molecular Genetics and Microbiology, Center for the Genomics of Microbial Systems, Duke University, Durham, North Carolina, United States of America; 2 Department of Cell Biology and Physiology, Center for Gastrointestinal Biology and Disease, University of North Carolina at Chapel Hill, Chapel Hill, North Carolina, United States of America; 3 Department of Developmental Biology, Stanford University, Stanford, California, United States of America; 4 Departments of Genetics and Biology, University of North Carolina at Chapel Hill, Chapel Hill, North Carolina, United States of America; 5 Department of Medicine, Center for Gastrointestinal Biology and Disease, University of North Carolina at Chapel Hill, Chapel Hill, North Carolina, United States of America; University of Pennsylvania, United States of America

## Abstract

The intestinal epithelium serves critical physiologic functions that are shared among all vertebrates. However, it is unknown how the transcriptional regulatory mechanisms underlying these functions have changed over the course of vertebrate evolution. We generated genome-wide mRNA and accessible chromatin data from adult intestinal epithelial cells (IECs) in zebrafish, stickleback, mouse, and human species to determine if conserved IEC functions are achieved through common transcriptional regulation. We found evidence for substantial common regulation and conservation of gene expression regionally along the length of the intestine from fish to mammals and identified a core set of genes comprising a vertebrate IEC signature. We also identified transcriptional start sites and other putative regulatory regions that are differentially accessible in IECs in all 4 species. Although these sites rarely showed sequence conservation from fish to mammals, surprisingly, they drove highly conserved IEC expression in a zebrafish reporter assay. Common putative transcription factor binding sites (TFBS) found at these sites in multiple species indicate that sequence conservation alone is insufficient to identify much of the functionally conserved IEC regulatory information. Among the rare, highly sequence-conserved, IEC-specific regulatory regions, we discovered an ancient enhancer upstream from *her6/HES1* that is active in a distinct population of Notch-positive cells in the intestinal epithelium. Together, these results show how combining accessible chromatin and mRNA datasets with TFBS prediction and in vivo reporter assays can reveal tissue-specific regulatory information conserved across 420 million years of vertebrate evolution. We define an IEC transcriptional regulatory network that is shared between fish and mammals and establish an experimental platform for studying how evolutionarily distilled regulatory information commonly controls IEC development and physiology.

## Author summary

The epithelium lining the intestine is an ancient animal tissue that serves as a primary site of nutrient absorption and interaction with microbiota. Its formation and function require complex patterns of gene transcription that vary along the intestine and in specialized intestinal epithelial cell (IEC) subtypes. However, it is unknown how the underlying transcriptional regulatory mechanisms have changed over the course of vertebrate evolution. Here, we used genome-wide profiling of mRNA levels and chromatin accessibility to identify conserved IEC genes and regulatory regions in 4 vertebrate species (zebrafish, stickleback, mouse, and human) separated from a common ancestor by 420 million years. We identified substantial similarities in genes expressed along the vertebrate intestine. These data disclosed putative conserved transcription factor binding sites (TFBS) enriched in accessible chromatin near IEC genes and in regulatory sites with accessibility restricted to IECs. Fluorescent reporter assays in transparent zebrafish showed that these regions, which frequently lacked sequence conservation, were still capable of driving conserved expression patterns. We also found a highly conserved region near mammalian and fish *hes1* sufficient to drive expression in a specific population of IECs with active Notch signaling. These results establish a platform to define the conserved transcriptional networks underlying vertebrate IEC physiology.

## Introduction

Epithelial cells lining the intestinal tract serve important and evolutionarily conserved functions in animal physiology. The intestinal epithelium is the primary site for absorption and metabolism of diverse dietary nutrients and xenobiotics, relays metabolic and immunological signals to the rest of the body, and provides a critical barrier to microorganisms that reside within the intestinal lumen [1]. Dysfunction in the development and physiology of intestinal epithelial cells (IECs) has been implicated in a growing number of human diseases, such as inflammatory bowel diseases [1], colorectal cancer [2], food allergy [3], obesity [4,5], malnutrition [6], and infectious diarrheas [7]. These insights have fueled considerable interest in the molecular and cellular mechanisms underlying IEC biology.

Due to the common evolutionary origins of the animal intestine, animal models are invaluable tools in understanding the intestinal epithelium, including its normal development and dysfunction. The appearance of a “through gut” with a distinct mouth, anus, and intermediate regions was an early step in bilaterian animal evolution [1]. It is thought that many of the anatomic and physiologic features of the intestine are conserved between bilaterian lineages, with mammals (members of Sarcopterygii) and bony fishes (members of Actinopterygii) last sharing a common ancestor approximately 420 million years ago [8]. Although lineages within these vertebrate taxa have evolved specific adaptations in their intestinal anatomy and physiology, fundamental aspects appear to be conserved [9]. For example, the intestinal epithelium in mammals and fishes comprises functionally similar IEC subtypes, including absorptive enterocytes and secretory cells such as goblet cells and enteroendocrine cells. These differentiated cells are rapidly renewed through the action of IEC stem or progenitor cells residing at the base of villi or rugae [10,11]. Another prominent conserved feature of the vertebrate intestine is anatomic and physiologic specialization along the anteroposterior axis. In mammals, the gut is generally composed of a small intestine, which includes a duodenum in which chemical digestion occurs, a jejunum in which the majority of nutrients are absorbed, an ileum that specifically absorbs bile salts and vitamin B12, and a colon or large intestine in which absorption of water and salts occurs. Though the intestinal tract of zebrafish and other fishes display anteroposterior regional specialization, the evolutionary relationship with mammalian intestinal regions has remained unclear. The zebrafish intestine was originally described to consist of 3 histologically defined segments: (1) anterior or rostral intestine, also known as the intestinal bulb or segment I; (2) the middle intestine or segment II; and (3) the posterior or caudal intestine or segment III [12–14]. Though this 3-segment nomenclature has been used to describe the zebrafish intestine from larval to adult stages, the extent to which intestinal segmental programs are maintained across zebrafish life stages remains unresolved. Transcriptomic characterization has shown that the anterior intestine of the adult zebrafish generally expresses genes with similar function to the mammalian small intestine, while the posterior zebrafish intestine corresponds to the mammalian large intestine [15]. However, it is generally unknown which gene sets are expressed in similar anteroposterior patterns across multiple species.

Recent studies have begun to identify transcription factors (TFs) and regulatory regions that contribute to the identity and function of IECs in individual species [16–19]. For example, CDX2 acts as a master sequence-specific TF that regulates intestinal patterning and epithelial identity in mice and zebrafish [18,20,21] and controls chromatin access to regulatory regions for other TFs that specify IEC identity in mammals, such as the small intestinal TFs HNF4A and GATA4 [18,22–24]. IEC subtype specification is similarly controlled through transcriptional regulatory mechanisms. These include Wnt signaling [25] and a Notch signaling cascade that uses downstream TFs such as RBPJ, ATOH1, and HES1, which direct IEC specification into secretory or absorptive lineages [12,17]. Still, it remains uncertain which portions of the regulatory framework defining IEC function are conserved, hindering the utility of model organisms to help dissect relevant signaling mechanisms, transcriptional programs, and disease states.

Genome-wide accessible chromatin assays can identify cell type and condition-specific cis-regulatory regions. These nucleosome-depleted regulatory regions contain transcription factor binding sites (TFBS) that provide a critical insight into the underlying transcriptional networks that define tissue identity. However, recent studies have found accessible regulatory regions largely similar when comparing IEC stem cells and their downstream subtype progenitor intermediates, in spite of differences in gene expression [17]. Similarly, gene expression changes induced in IECs upon colonization with a microbiota were not associated with overt alterations in the accessible chromatin landscape [16]. Together, these findings suggest that aspects of IEC cell plasticity, differentiation, and environmental response are not driven by gross changes in the accessible chromatin landscape, making key regulatory regions difficult to identify. Differential expression or binding of lineage-specific or environmentally responsive TFs [16,26] or chromatin modifiers like the histone deacetylase HDAC3, which plays important roles in how IECs respond to microbes [27], may partially explain the lack of gross chromatin accessibility changes in certain IEC populations [16,17]. However, it remains unclear which regulatory mechanisms serve similar roles in IEC function in different species or if conserved accessible chromatin regions across species can identify important regulatory mechanisms that have not been easily identified within a single species [28].

In this study, we tested the hypothesis that conserved IEC functions are achieved using conserved transcriptional regulatory mechanisms. We profiled the transcriptome and accessible chromatin landscape of IECs from 4 evolutionarily distant vertebrates: zebrafish, stickleback, mouse, and human. We found substantial overlap at a transcriptional level including a common group of IEC signature genes, evidence for common regulation of IEC subtype specification, and unexpected similarity between gene expression along the length of the intestine from fish to mammals. These transcriptional similarities were not easily explained by neighboring conserved regions that were commonly accessible in IECs. However, using accessible chromatin regions and TFBS prediction we were able to recover common IEC-related regulatory information genome wide and at several important representative loci, despite a scarcity of sequence conservation around genes commonly expressed in IECs for over 420 million years.

## Results

### Intestinal epithelial gene expression is conserved in vertebrates

**Fig 1:**
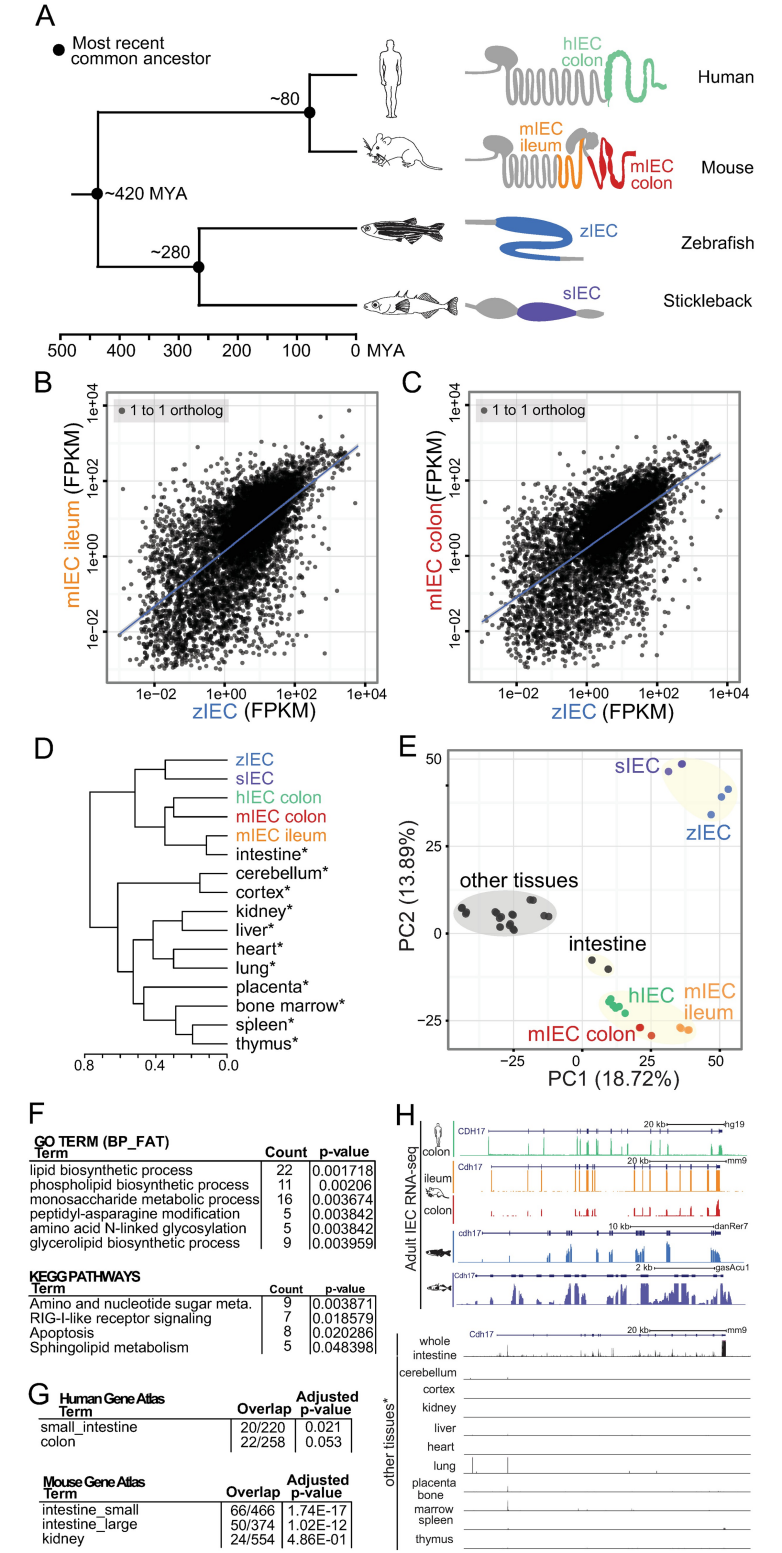
Transcriptional profiling of intestinal epithelial cells (IECs) from multiple species show conserved expression after 420 million years since a common ancestor. **(A)** (Left) A phylogenetic tree showing time since a common ancestor for human (*Homo sapiens*), mouse (*Mus musculus*), zebrafish (*Danio rerio*), and stickleback (*Gasterosteus aculeatus*) species. (Right) Simplified schematics showing the intestinal tract of all 4 organisms in gray with the region of collected IEC sample colored: green (human colon IECs), orange (mouse ileum IECs), red (mouse colon IECs), blue (zebrafish whole intestine IECs), and purple (stickleback whole intestine IECs). **(B)** Scatterplot of Fragments Per Kilobase of transcript per Million mapped reads (FPKM) values for 1-to-1 orthologs from mouse ileum IEC and zebrafish IEC samples shows a positive correlation coefficient (Pearson *r*^2^ = .273; Spearman *r*^2^ = .416). **(C)** Same as (B) for mouse colon IECs and zebrafish IECs (Pearson *r*^2^ = .341; Spearman *r*^2^ = .379). **(D)** Complete linkage cluster analysis using FPKM values for 1-to-1 orthologs show similarity between mRNA levels for IECs in comparison to other mouse tissues. Scale represents values of Pearson distance. Black data sets marked by asterisks are RNA sequencing (RNA-seq) experiments from non-IEC tissues [29]. **(E)** Scatterplot of principal component 1 and 2 (PC1 and PC2) using principal component analysis (PCA) of FPKM values for all IEC data sets and other mouse tissues. **(F)** Top reported Gene Ontology (GO) terms and Kyoto Encyclopedia of Genes and Genomes (KEGG) pathways from Database for Annotation Visualization and Integrated Discovery (DAVID) using IEC signature genes. The enrichment of these gene groups do not clear Bonferroni correction thresholds (S2 Table). **(G)** Top tissues showing overlap with IEC signature genes from human and mouse Gene Atlas using Enrichr. **(H)** The University of California Santa Cruz (UCSC) screenshot of RNA-seq levels at cadherin 17 (*CDH17*) in 4 species’ IECs (top) and other tissues (bottom) [29] shows expression largely restricted to IECs.

In order to understand the extent of gene expression similarity across vertebrate intestinal epithelia, we compared newly generated gene expression data from IECs isolated from adult human colon, adult zebrafish intestine, adult stickleback intestine, and from data we previously generated from adult mouse colon and ileum [16] (Fig 1A). We find a strong correlation in gene expression between fish and mammalian IECs throughout the dynamic range of the transcriptome (Fig 1B and 1C, S1A Fig). Using principal component analysis (PCA) and hierarchical clustering, we find that the expression of orthologous genes is more similar amongst IECs across species than mouse IECs are to other mouse tissues (Fig 1B–1E, S1 and S2 Figs). Furthermore, unrelated RNA sequencing (RNA-seq) data from whole mouse intestine cluster with data from all vertebrate IECs (Fig 1D and 1E) [29]. These results reveal that gene expression levels in IECs are similar across these 4 vertebrate species and suggest that many aspects of IEC physiology have been conserved since the common ancestor of mammals and fish.

In our PCA of mRNA expression from IECs and mouse tissues, we found that principal component 1 (PC1) separated mammalian and fish IECs from all other tissues. We identified 470 genes whose expression levels highly correlate with PC1 and exhibit high expression in IECs relative to other tissues, though their expression and function is not necessarily exclusive to IECs (Fig 1D and 1E, S1 Fig, S1 Table, Materials and methods). These IEC signature genes are representative of physiologic functions and cell types in the intestinal epithelium and include genes involved in lipid, carbohydrate, and protein metabolism (Fig 1F and 1G, S2 Table). IEC signature genes, including retinol binding protein 2 (*RBP2*, with *rbp2a* assigned as the zebrafish ortholog by Ensembl), fatty acid binding protein 6 (*FABP6*), and cadherin 17 (*CDH17*), are amongst the most highly expressed genes in IECs from several species (Fig 1H, S1D Fig). In addition, we identified genes indicative of different IEC subtypes within the intestinal epithelium including *RBP2* [30] and *FABP6* (enterocyte) [31], peptide YY (*PYY*; enteroendocrine) [32], and polypeptide N-acetylgalactosaminyltransferase 6 (*GALNT6*; goblet cells) [33], consistent with our RNA-seq data representing heterogeneous populations of IECs (S1D Fig). Ribosomal protein genes and translation components were also highly represented within this signature, consistent with the intestinal epithelium being one of the most highly-proliferative tissues [33]. Amongst the IEC signature genes, we found TFs known to be involved in development and function in the intestine, including the epithelial-specific E26 transformation-specific (ETS) TF *ELF3* [34], *HNF4A* [18], *HNF4G* [35], *FXR* [36], *GATA5* [37], and *OSR2* [38], suggesting a conserved basal IEC transcriptional network. Several IEC signature TFs have known associations with human Inflammatory Bowel Diseases (IBD), including *SMAD7* [39], *CEBPG* [40], *STAT3* [40–42], *XBP1* [43], *HNF4A* [44], *ELF3* [41], *IRF1* [45], and NFκB components *IKBKB*, *IKBKG*, and *NFKBIZ* [46]. Furthermore, the most common human disorders associated with IEC signature genes were obesity-related traits, IBD, and Type 2 Diabetes (S1 Table). Together these data highlight the utility of mouse, zebrafish, and stickleback in modeling human intestinal development and disease and suggest a basal similarity in the transcriptional mechanisms underlying intestinal epithelial homeostasis in vertebrates.

### Evidence for transcriptional conservation and regulation along the length of the intestine

**Fig 2:**
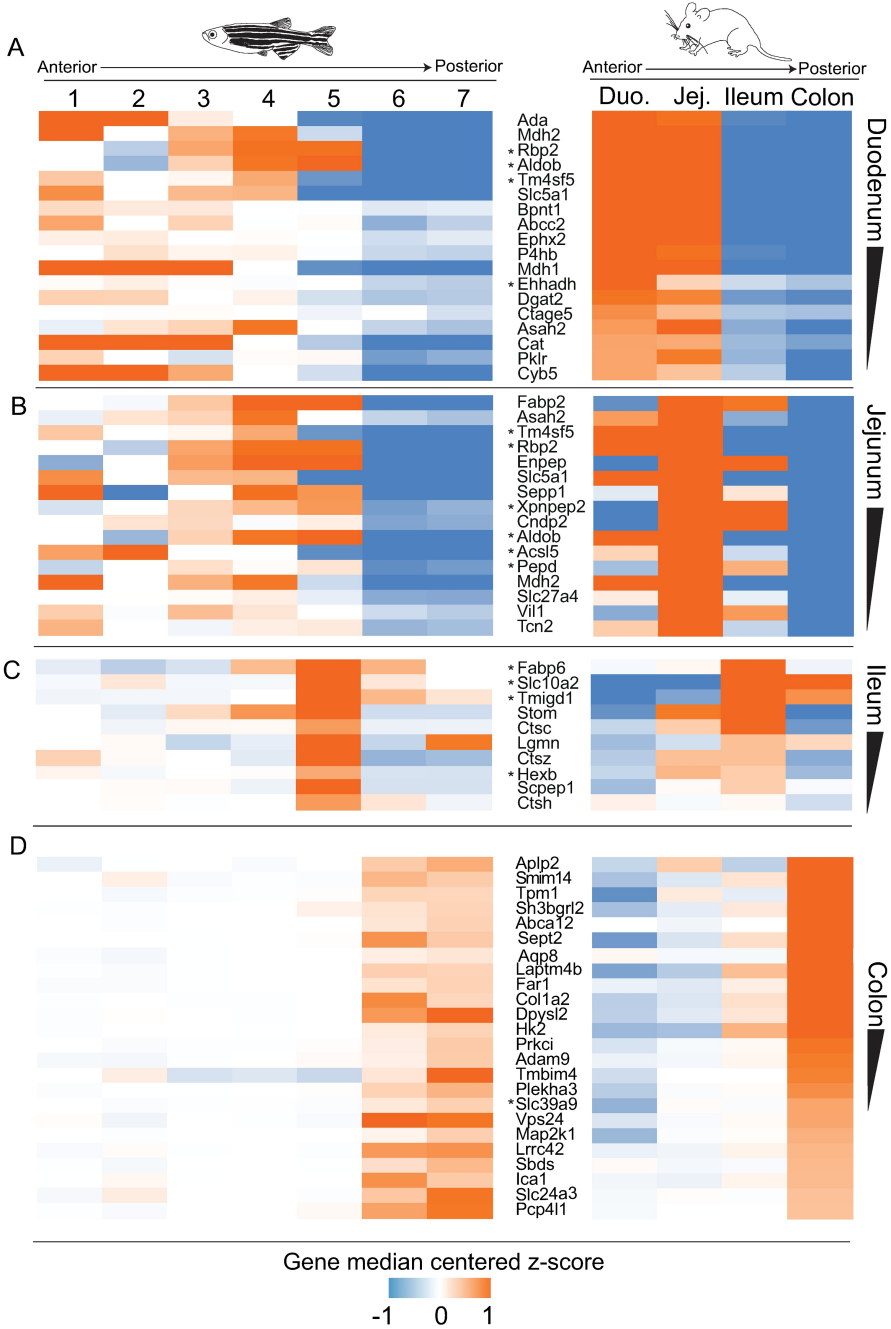
Identification of genes with conserved regional transcriptional specification along the length of the intestine in zebrafish and mouse. **(A)** A heat map comparing gene median-centered z-scores of 1-to-1 orthologs from previously published datasets profiling expression levels along the length of the zebrafish [15] and mouse intestine (Materials and methods, S3 Fig) [47]. Similarly expressed genes are ordered by expression values of mouse **(A)** duodenum, **(B)** jejunum, **(C)** ileum, and **(D)** colon. Gene names with asterisks are also intestinal epithelial cell (IEC) signature genes as defined in Fig 1. Genes can exist in multiple subfigures.

The results described above revealed conserved IEC signatures by comparing RNA levels from intestinal IECs to other tissues. However, we speculated that signatures of intestinal identity might be further resolved by comparing gene expression along the intestine’s anteroposterior axis in zebrafish and mice. Using previously published datasets we identified gene orthologs that showed similar expression patterns along the adult zebrafish intestine (divided into 7 sections of equal length) [15] and along the adult mouse intestine (divided into duodenum, jejunum, ileum, and colon) (Fig 2, S3 Fig, Materials and methods) [47]. Of 493 genes that showed high expression in the anterior of the zebrafish intestine, we found over 70 genes sharing similar high expression in the anterior intestine of mouse [15], including IEC signature genes like *Rbp2*, *Aldob*, and *Ehhadh* (Fig 2A and 2B, S3 Fig). Many of these genes (e.g., *Fabp2*, *Acsl5*, *Agpat2*, *Slc27a4*, and *Dgat2*) are critical in lipid metabolism and uptake, which is consistent with lipid absorption and metabolism taking place mostly in the small intestine in mouse and anterior portion of the intestine in zebrafish [48]. However, ordering genes by mouse duodenum expression level reveals that some of these genes have surprisingly high similarities in cross-species expression patterns along the length of the intestine (Fig 2A and 2B, S3C Fig). For example, adenosine deaminase (*Ada*), which is most highly expressed in duodenum in mouse [49], is most highly expressed in the zebrafish sections 1–2 (Fig 2B, S3C Fig). Similarly, *Fabp2* and *Enpep*, which are expressed most highly in the jejunum and ileum in mouse, are most highly expressed in sections 3–5 in zebrafish intestine (Fig 2C and S3C Fig). This suggests an unappreciated similarity between IEC gene expression along the small intestine in mammals (mouse) and teleosts (zebrafish) and the potential for further examples of subregionalization in the zebrafish intestine that have not been previously described [13–15].

In support of this conserved regional specification, we also found a small group of genes that are expressed highly only in the terminal portion of the zebrafish anterior intestine (section 5 [15]), which occupies a location similar to the mammalian ileum (Fig 2C and S3D Fig). The mammalian ileum is involved in the uptake of bile salts following their use in emulsification of lipids in the anterior small intestine [50]. Two IEC signature genes involved in bile handling, *Fabp6* and *Slc10a2*, show high expression in this narrow region of zebrafish intestine and mouse ileum (Fig 2C and S3D Fig). In addition, the proteases *Lgmn*, *Scpep1*, and 3 cathepsins are in this cluster and show similar high expression largely in the mouse ileum, suggesting a regionally conserved utilization of lysosomal-cathepsin—mediated degradation (Fig 2C and S3D Fig) [51]. These observations suggest that the cellular differentiation and physiological programs deployed in this region of the zebrafish intestine are specialized for bile salt recovery, with strong homology to the mammalian ileum. We also found numerous genes expressed more highly in the posterior end of the mouse and zebrafish intestine, suggesting similar physiologic functions in zebrafish distal intestine and mouse colon (Fig 2D, S3E and S3F Fig) [15]. Collectively, these observations suggest that the teleost intestine has the capacity to articulate complex gene differentiation patterns along the length of the intestine that are functionally and spatially analogous to segments in the evolutionarily distant mammalian intestine.

### Accessible chromatin profiling of IECs in 4 species identifies putative IEC regulatory regions

**Fig 3:**
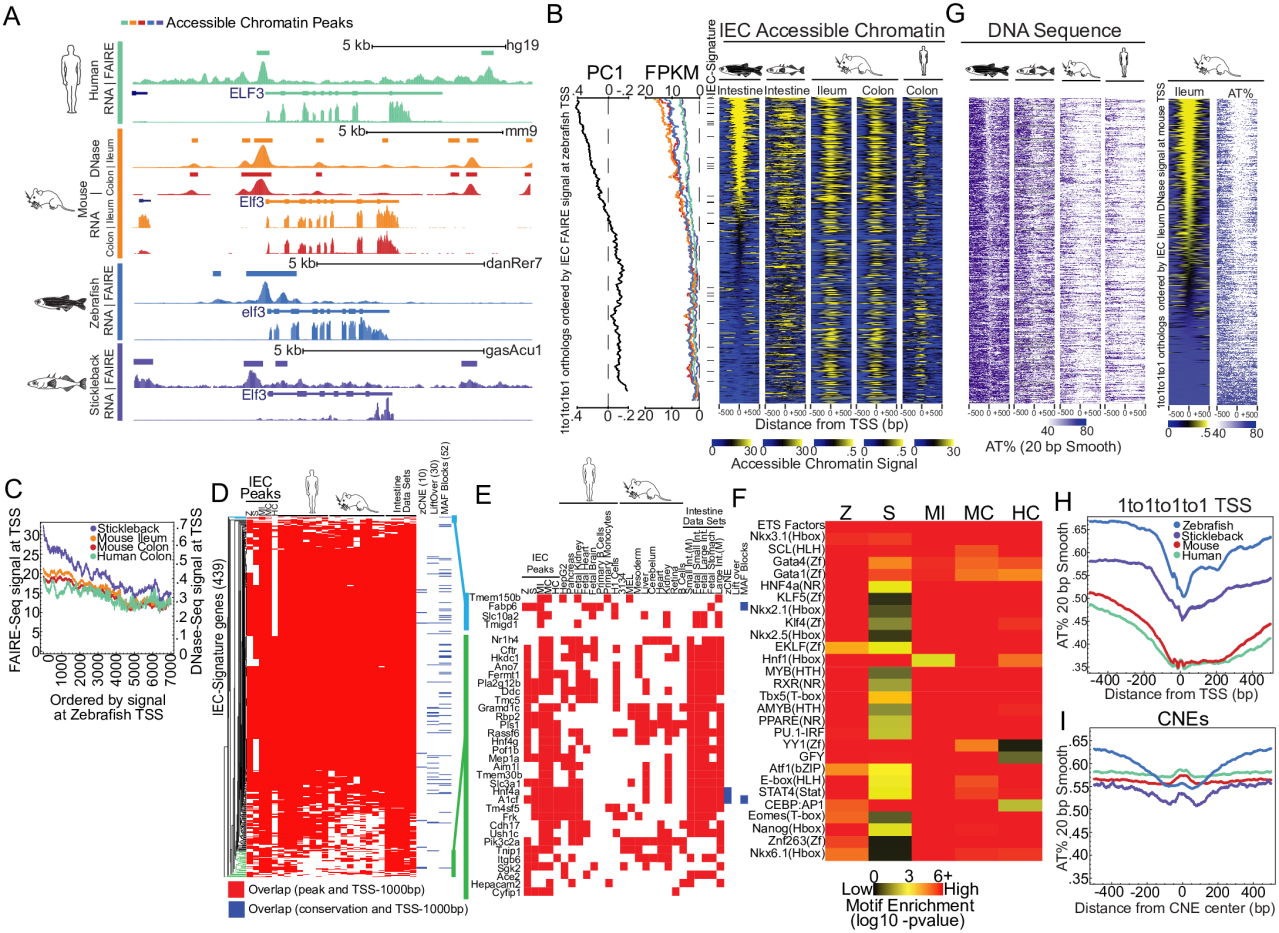
Accessible chromatin maps in intestinal epithelial cells (IECs) from multiple species reveals common regulatory information without substantial sequence conservation. **(A)** Accessible chromatin and RNA sequencing (RNA-seq) data from representative replicates for the IEC signature gene *ELF3* for each organism. For each organism, gene models are represented with thick bars (exons) and thin bars (introns and untranslated regions). **(B)** Accessible chromatin signal at the 1,000 bp surrounding the transcription start site (TSS) of 1-to-1-to-1-to-1 orthologs ordered by zebrafish IEC Formaldehyde-Assisted Isolation of Regulatory Elements sequencing (FAIRE-seq) signal at the base pair coordinate of the gene’s TSS for zebrafish, stickleback, mouse ileum, mouse colon, and human colon (Right). Moving medians (Left) are shown for PC1 correlation value used to identify IEC signature genes (500 gene window, 1 gene step; black), Fragments Per Kilobase of transcript per Million mapped reads (FPKM) of associated genes (250 gene window, 1 gene step; color scheme based on data sets presented in A and throughout), and IEC signature genes are marked by black horizontal bars. **(C)** Moving median of accessible chromatin signal at TSS of stickleback, mouse ileum, mouse colon, and human ordered by signal at zebrafish TSS (250 gene window, 1 step) highlight the relationship between IEC accessible chromatin data in multiple species. Numerical values can be found in S1 Table. **(D)** A heatmap of cluster analysis of overlap between accessible chromatin peak calls for IEC samples and additional published accessible chromatin datasets with the region 1,000 bp upstream of the TSS (TSS-1,000 bp) for IEC signature genes (red bars). Overlap between TSS-1,000 bp and conservation metrics are represented with blue bars. The total number of overlaps is represented in parentheses for each conservation metric column, and overlaps are defined as having at least 1 shared base pair. Initials are used to specify IEC data sets: Z; Zebrafish, S; Stickleback, MI; Mouse Ileum, MC; Mouse Colon, HC; Human Colon. **(E)** Closeup of the heatmap of clustered genes from panel 3D that frequently showed putative conserved accessible chromatin at TSS in IEC samples (light blue and green). Color scheme is shared with 3D. **(F)** A heatmap highlighting common transcription factor binding sites (TFBS) motif enrichment within the accessible chromatin peaks that fall between the region 10,000 bp upstream of the TSS and 10,000 bp downstream of the transcription termination site (TTS) of IEC signature genes. Motif enrichment includes known transcription factors (TFs) involved in IEC biology which are also often expressed highly in our IEC samples. E26 transformation specific (ETS) factors include motifs for ELF5, EHF, ELF1, GABPA, ETS, Elk1, Fli1, Elk4, ETS1, ERG, SPDEF, and EWS:FLI. **(G)** The percentage of DNA bases that are either adenosine or thymine (AT%) (20 bp smooth: 20 bp window, 1 bp step) surrounding TSS as ordered by IEC zebrafish FAIRE signal from (B) and IEC mouse Ileum DNase signal (Right) shows nonrandom patterns at TSS that **(H)** vary on average from species to species. **(I)** Comparison of AT% (20 bp smooth) at conserved nonexonic elements (CNEs) that by their nature show some similar sequence composition.

To test the hypothesis that conserved regions of accessible chromatin underlie the transcriptional similarities we found in IECs, we profiled accessible chromatin in the same cell preparations that we used to generate our RNA-seq data using Formaldehyde-Assisted Isolation of Regulatory Elements sequencing (FAIRE-seq) from stickleback, zebrafish, and human colon IECs. We also used recently published data from our group that profiled mouse ileum and colon IECs using DNase I hypersensitive sites sequencing (DNase-seq) [16]. Combining IEC accessible chromatin maps with species-transferable regulatory landmarks such as the transcription start site (TSS) and conserved nonexonic elements (CNEs) allowed us to profile and define common regulatory information utilized in all species. Accessible chromatin peaks were frequently enriched at orthologous TSS in IECs, including at IEC signature genes such as *ELF3* (Fig 3A, S3 Table and S2 Fig). This accessible chromatin signal is consistent with typical genome-wide distributions, though the relationship with accessibility may not be strictly driven by regulatory regions or transcription that is specific to IECs (Fig 3B and 3C). However, the related IEC PC1 correlation values, transcription levels, and presence of IEC signature genes were both higher on average at the TSS with higher accessible chromatin levels, suggesting that the magnitude or presence of accessible chromatin may be conserved at related regulatory regions in IECs in distantly related species (Fig 3B and 3C, S4A and S4B Fig).

We compared accessible TSS call overlaps at the TSS for all IEC samples and several cell lines and tissues from mouse and human Encyclopedia of DNA Elements (ENCODE)/Roadmap [52–54] to determine if accessible chromatin status could identify IEC-specific regions. We clustered this information to identify common patterns of accessible chromatin status in all these samples (Fig 3D). While the majority of TSS regions appeared to be constitutively accessible at most genes in most species and tissues represented, a group of IEC signature genes had TSSs that were accessible frequently in IECs but less often in other tissues (Fig 3E). Importantly, genes within this group were also almost always accessible at the TSS in several independent datasets of intestinal tissue from mouse and human (Fig 3E). This group included key genes involved in IEC biology such as *HNF4A*, *HNF4G*, *RBP2*, *A1CF*, *CFTR*, and *CDH17*. Further, we found 3 genes, *FABP6*, *SLC10A2*, and *TMIGD1*, that showed high similarity of mRNA levels along the length of the intestine at the mouse ileum and zebrafish section 5 (Fig 2C) and showed limited accessibility in nonintestinal tissues with accessibility in some of the intestine-related datasets (Fig 3E).

To determine if the regions that show chromatin accessibility largely in IECs also showed conservation at a sequence level, we used multiple metrics that measure sequence conservation from teleost to mammals. This included zebrafish conserved nonexonic elements (zCNEs), a stringent comparison of noncoding regions of 14 species, including human and mouse [55]. We also used UCSC liftOver and zebrafish-to-human and zebrafish-to-mouse multiple alignment format (MAF) blocks to identify potentially DNA-conserved regions. Though metrics at the RNA and chromatin levels suggest substantial similarities between teleost and mammalian intestine and these TSS regulatory regions often show sequence constraint at some level, less than 15% of IEC signature genes had detectable conservation from zebrafish to mouse or human in TSS regions. This hampered our ability to infer which regulatory regions and putative TFBS are actually conserved across these species (Fig 3E) [56]. Additionally, most of these conserved regions were identified by MAF blocks and appeared to have only small regions of highly degenerate sequence conservation from teleosts to mammals that were located immediately upstream of transcribed regions and suggestive of minimal functional conservation. We speculated that common regulatory information could still be shared in these TSS regions because short, modular TFBS could escape sequence conservation metrics [56–58]. We looked for enrichment of TFBS using a library of 303 position weight matrices (PWMs) (primarily derived from human ChIP-seq datasets) within 2 sets of accessible chromatin peaks: (1) those between 10 kb upstream of the TSS to 10 kb downstream of the transcription termination site (TTS) [59] (Fig 3F) and (2) those at the TSS of IEC signature genes (S4C and S4D Fig). Common enrichment of PWMs in accessible chromatin regions at IEC signature genes of different species identified several TF motifs known to regulate IEC expression in mammals including HNF1, HNF4A, GATA4, KLF5, and the often similar ETS factors, including ELF3 and SPDEF (Fig 3F, S4C and S4D Fig) [60–63], that may represent a TF network that control core conserved aspects of IECs in animals.

Conservation metrics rely on relatively long stretches of DNA sequence, so we tried to identify sequence properties that varied between species and might interrupt the maintenance of easily detected conserved sequence. By comparing the percentage of DNA bases that are either adenosine or thymine (AT%) at TSSs ordered by zebrafish FAIRE signal, we found that gross sequence characteristics are substantially different at the TSS of orthologs between species (Fig 3G and 3H). AT% decreases at the TSS from approximately 51% in zebrafish to 46% in stickleback to 35% in mouse and human (Fig 3H). These differences are maintained on average in the area surrounding the TSS (Fig 3G and 3H). This general phenomenon likely has a substantial influence on the maintenance of sequence at these regulatory regions [64,65] and may represent a particular challenge for the identification of conserved regions and regulators. However, the substantial difference in AT% observed at TSS is absent on average at CNEs, which, based on the method that they are identified, are inherently similar in sequence (Fig 3I). This suggests that general sequence utilization differences seen at TSSs in different species on average are important but may not influence all regulatory regions.

### Conservation of regulators that define regional intestinal expression from fish to mammals without apparent sequence conservation

The apparent maintenance of TFBS enrichment suggested that our accessible chromatin and RNA-seq data were identifying functionally conserved regions, although they frequently lacked conserved DNA sequence. To test this, we cloned regions upstream of the *rbp2a* and *fabp6* TSS that showed no substantial sequence conservation from fish to mammals, but appeared accessible largely in IECs, and tested them using a functional in vivo zebrafish reporter assay (Materials and methods). Because both *RBP2* and *FABP6* are IEC signature genes that showed strong regional conservation of expression in zebrafish and mouse (Fig 2), this allowed us to simultaneously test if the conservation of regulatory information was interpreted by zebrafish to specify intestinal regionality in addition to IEC expression generally.

**Fig 4:**
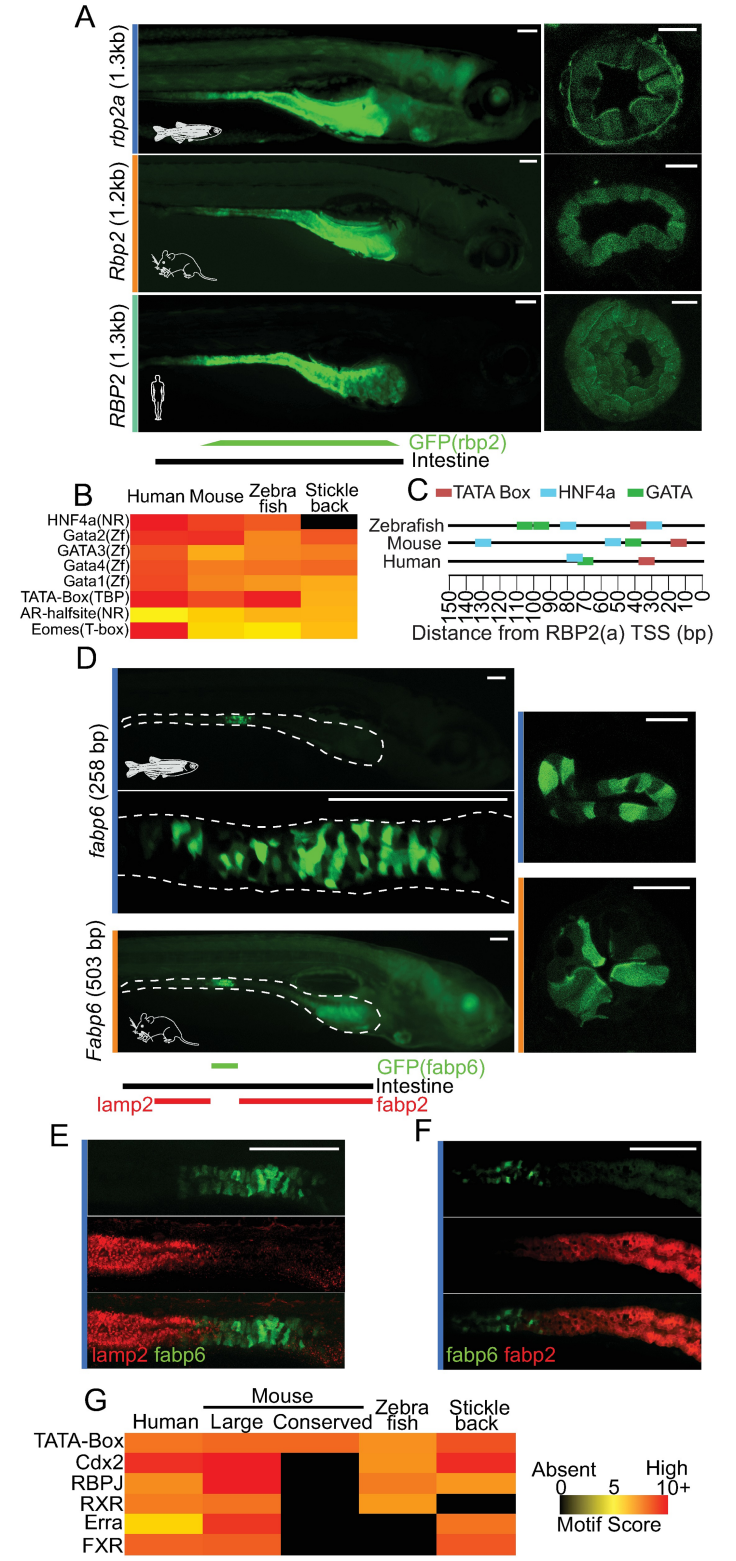
Exogenous regulatory regions identified by intestinal epithelial cell (IEC) accessible chromatin can drive regionally conserved IEC expression in zebrafish larvae. **(A)** (Left) Whole-mount stereofluorescence images of 7 dpf zebrafish stable lines expressing a green fluorescent protein (GFP) reporter construct for *rbp2a/Rbp2/RBP2* accessible regulatory regions from zebrafish, mouse, and human show high levels of expression in IECs in the anterior portion of the intestine. Scale bar 100 μm. (Right) Representative cross-sections of zebrafish intestine confirming high IEC expression for each corresponding stable line. The zebrafish cross-section was taken in the posterior of the intestinal bulb, and the mouse and human cross-sections were taken within the middle of the intestinal bulb. Scale bar 25 μm. **(B)** Common motifs detected in the *RBP2*(*a*) cloned region for each species colored by Homer motif scores. Motif score scale is shared with Figure 4G. **(C)** Schematic of common transcription factor binding sites (TFBS) motifs found immediately upstream of the transcription start site (TSS) of *RBP2(a)* in zebrafish, mouse, and human. **(D)** (Left blue) Whole-mount stereofluorescence images of 7 dpf zebrafish stable line *Tg*(*fabp6*:*GFP*) show high levels of GFP expression in IECs in the middle of the intestine. (Below blue) Average projection of confocal stacks of whole-mount zebrafish *Tg*(*fabp6*:*GFP*). Scale bar 100 μm. (Right blue) Representative cross-section of zebrafish intestine confirming high IEC expression for *Tg(fabp6*:*GFP)*. Scale bar 25 μm. (Left orange) Whole-mount stereofluorescence image of 7 dpf zebrafish stable line *Tg(Mmu*.*Fabp6*:*GFP)* shows high levels of GFP expression in IECs in the middle of the intestine. Scale bar 100 μm. (Right orange) Representative cross-section of zebrafish intestine confirming high IEC expression for *Tg(Mmu*.*Fabp6*:*GFP)*. Scale bar 25 μm. A smaller conserved region from mouse and 2 inclusive regions from human *FABP6* did not drive IEC GFP expression in our zebrafish reporter assay. Throughout, the white dashed line marks the boundary of intestine and IECs. **(E)** Confocal whole-mount maximum projection image showing lack of overlap between *Tg(fabp6*:*GFP)* and the segment 2 marker *TgBAC(lamp2-RFP)*. Scale bar 100 μm. **(F)** Confocal whole-mount average projection image showing lack of overlap between *Tg(fabp6*:*GFP)* and the segment 1 marker *Tg(-4*.*5fabp2*:*DsRed)*. Scale bar 100 μm. **(G)** Common motifs detected in the cloned or corresponding region for each species colored by Homer motif score.

We generated a transgenic reporter construct with the 1.3 kb region upstream of the TSS of zebrafish retinol binding protein gene, *rbp2a*, cloned upstream of the mouse *cFos* minimal promoter and green fluorescent protein (GFP) [*Tg(rbp2a*:*GFP)*] (Figs 4A and 3E, S5A, S6A Figs and S4 Table). *Tg(rbp2a*:*GFP)* was capable of driving high expression in the anterior portion of the intestinal epithelium in larvae (Fig 4A**)**, which was consistent with the known expression patterns of *rbp2a* [15,66,67], suggesting that sufficient regulatory information to drive regional expression in IECs is contained within this fragment. This expression pattern is distinct from a general control reporter construct in which no additional DNA was cloned upstream of the mouse *cFos* minimal promoter driving GFP, and no consistent IEC expression is found (S6B–S6I Fig) [68]. Using the same reporter assay, we then tested the region immediately upstream of *Rbp2* from mouse *Tg(Mmu*.*Rbp2*:*GFP)* and *RBP2* from human *Tg(Hsa*.*RBP2*:*GFP)* and found that both were capable of driving GFP in IECs in the anterior portion of the larval zebrafish intestine (Fig 4A and S5A Fig). We tested if we could identify common, small TFBS that might have escaped detection by conservation metrics in these regions but explain the conserved patterns of IEC expression. TF motif searching within the *RBP2* fragments for zebrafish, mouse, and human identified shared strong matches to HNF4A and GATA factors within a few hundred bases of the TSS (Fig 4B and 4C). To test how often HNF4A and GATA motifs occurred generally, we queried a similarly sized area 150 bp upstream of the TSS of all genes. Only .23% (79 genes), .25% (51 genes), and .25% (47 genes) had both HNF4A and GATA4 motifs in zebrafish, mouse, and human, respectively, and no other 1-to-1-to-1 orthologs contained both HNF4A and GATA4 motifs at this location in all 3 species. This suggests that the presence of HNF4A and GATA sites in the *RBP2* TSS is conserved among vertebrate genomes and is unlikely to have occurred by chance. Indeed, HNF4A and GATA4 are likely a common vertebrate regulatory cassette as studies in multiple species have identified their importance in small intestinal IEC biology (S3G and S3H Fig) [26,69]. Collectively, these results establish that accessible chromatin maps can help discern conserved motif information and how in vivo reporter assays can be used to test for potential conserved tissue-specific regulatory activity.

### Fabp6 regulatory region drives conserved expression in a region of the zebrafish intestine positionally and functionally analogous to the mammalian ileum

We next sought to interrogate the regulatory potential of regions upstream of *FABP6*, an IEC signature gene expressed primarily in the ileum in mouse and human and whose TSS was accessible in all IECs tested except mouse and human colon (Figs 3E and 4D). The region 258 bp upstream of the zebrafish *fabp6* TSS [*Tg(fabp6*:*GFP)*] drove a very specific GFP expression domain exclusively in IECs in the middle of the larval zebrafish intestine, consistent with the endogenous pattern of *fabp6* mRNA expression (Figs 2C and 4D, S5B and S5G–S5J Fig) [15,47,70]. Unlike *rbp2a*, *fabp6* had a small region upstream of the *fabp6* TSS that was conserved to mouse and human. However, this appeared to only correspond to a TATA-box (Fig 4G, S5B and S5C Fig), and cloning of this small region from mouse to test in our zebrafish reporter assay did not drive expression in IECs. However, when we included the entire accessible chromatin region from mouse *Tg*(*Mmu*.*Fabp6*:*GFP)* (a 503 bp region upstream of mouse *Fabp6* TSS), which included the minimal conserved region, we found this larger sequence sufficient to drive an IEC expression pattern that was positionally identical to the corresponding region from zebrafish (Fig 4D). This suggests the regulatory information necessary to drive IEC expression in the putative zebrafish ileum is within the additional region defined by accessible chromatin from mouse and not solely detected by conservation.

In order to define the relationship between this *fabp6* domain in the context of the canonical 3 segments of the zebrafish intestine [14], we compared the larval expression pattern of *Tg*(*fabp6*:*GFP*) with zebrafish segment 2 marker *TgBAC(lamp2-RFP)* [71] and intestinal segment 1 marker *Tg(-4*.*5fabp2*:*DsRed)* [72,73]. Strikingly, we found *Tg*(*fabp6*:*GFP*) did not overlap with either, suggesting the intestinal region marked by *Tg*(*fabp6*:*GFP*) is a novel distinct segment of the zebrafish larval intestine (Fig 4E–4F). Similar regions upstream from human *FABP6* were negative for driving expression in zebrafish IECs, despite the putative presence of shared IEC-related TFBS like CDX2, RBPJ, and RXR (Fig 4G, S5B Fig and S4 Table). The combined evidence of conserved positional expression of *Fabp6* and other ileal genes (Fig 2C and S3D Fig) together with the maintenance of region-specific cis-regulatory information at zebrafish and mouse *Fabp6* orthologs (Fig 4D–4G) indicate that an intestinal segment functionally and regionally homologous to mammalian ileum is maintained in zebrafish larvae and likely specified by similar regulators.

### Transcriptional domains identified in larval zebrafish intestine are maintained in adults

**Fig 5:**
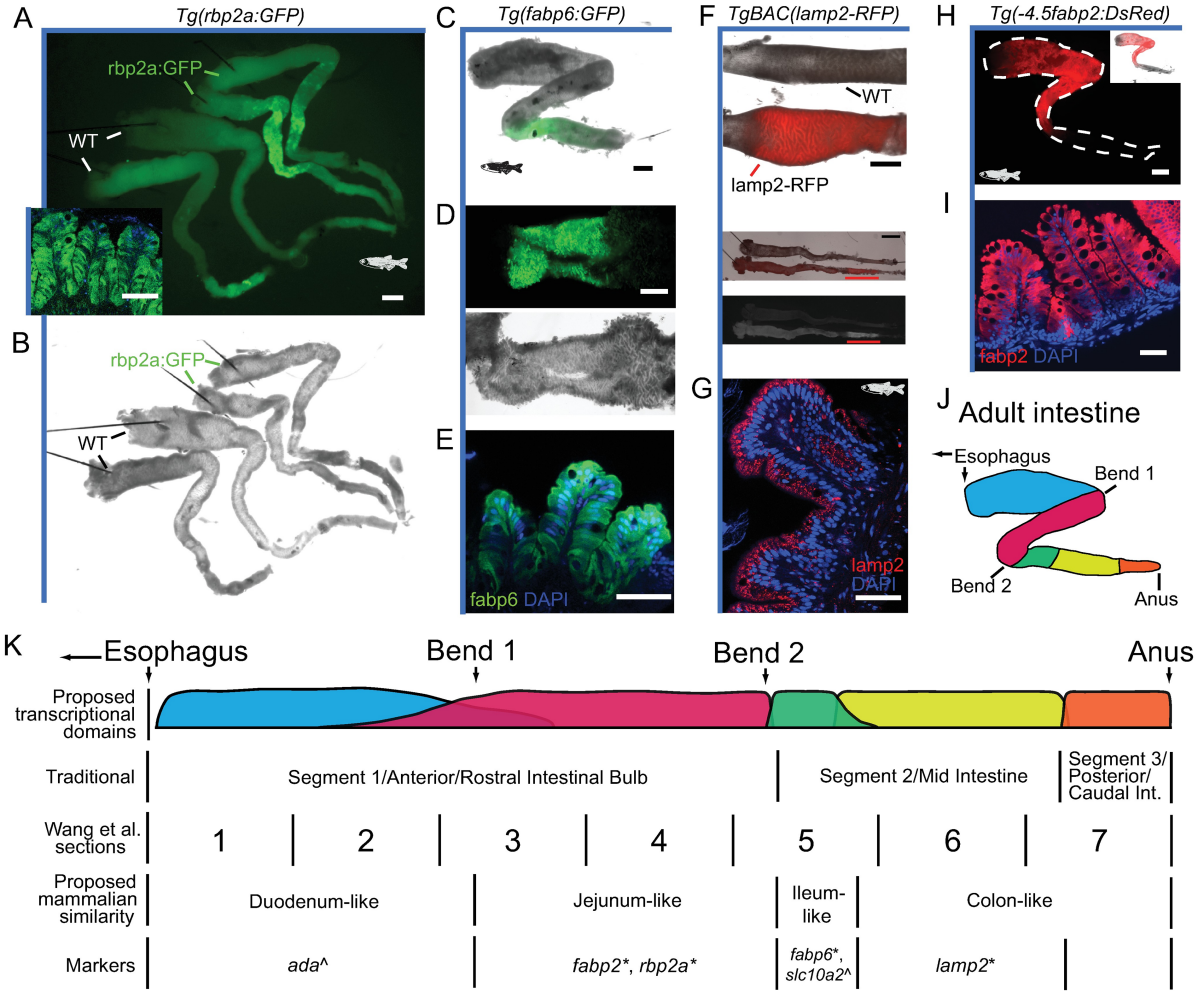
Transcriptional domains identified in larval zebrafish intestine are maintained in adults. **(A)** Stereomicroscopy image of 2 dissected adult *Tg(rbp2a*:*GFP)* intestines (top) and 2 wild-type (WT) intestines without green fluorescent protein (GFP) (bottom) shows high GFP expression between the first and second bend of the adult intestine in *Tg(rbp2a*:*GFP)*. Autofluorescence can be seen in several intestines following the second bend, presumably due to bile or feces (see brightfield microscopy in B). Scale bar 1,000 μm. Confocal cross-section of adult *Tg*(*rbp2a*:*GFP*) intestinal folds with DAPI in blue shows GFP expression in intestinal epithelial cells (IECs) in the inset panel. Scale bar 50 μm. **(B)** Brightfield microscopy of 2 dissected adult *Tg(rbp2a*:*GFP)* intestines (top) and 2 wild-type intestines (bottom). Dissection pins can be seen as long black lines anchoring the intestine in the intestinal bulb. **(C)** Stereomicroscopy image of brightfield and GFP overlay for the dissected intestine of adult *Tg*(*fabp6*:*GFP*), showing GFP expression in a discreet domain immediately following the second bend. The anus was excluded in this preparation. Scale bar 1,000 μm. **(D)** Stereomicroscopy image of GFP (top) and brightfield (bottom) for the dissected adult intestine of *Tg*(*fabp6*:*GFP*) opened lengthwise to show internal detail of the discreet domain of GFP expression. Scale bar 1,000 μm. **(E)** Confocal cross-section of adult *Tg*(*fabp6*:*GFP*) intestinal folds with DAPI in blue shows high GFP expression in IECs. Scale bar 50 μm. **(F)** Stereomicroscopy close-up of red fluorescent protein (RFP) and brightfield for adult WT and *TgBAC(lamp2-RFP)* zebrafish lines (top) showing entire intestines with the segment 2 relative position marked with a red horizontal line (middle). Scale bar 2,000 μm. Fluorescence-only image showing the distribution of the lamp2-RFP signal, highest in segment 2 (bottom). The relative orientation of the WT (top) and *TgBAC(lamp2-RFP)* (bottom) lines is maintained throughout. **(G)** Confocal cross-section of adult *TgBAC(lamp2-RFP)* intestinal folds from segment 2 with DAPI in blue shows high lamp2-RFP expression in IECs. Scale bar 50 μm. **(H)** (Top) Stereomicroscopy image for a dissected intestine of adult *Tg(-4*.*5fabp2*:*DsRed)* showing DsRed expression most highly between the first and second bend with additional expression in the intestinal bulb. The white dotted line references the dissected intestine. A similar *Tg(fabp2*:*RFP)* result has previously been reported [15]. Scale bar 1,000 μm. Overlay between DsRed and brightfield is shown in the inset in the upper right. **(I)** Confocal cross-section of adult *Tg(-4*.*5fabp2*:*DsRed)* intestinal folds with DAPI in blue shows high DsRed expression in IECs. Scale bar 50 μm. **(J)** Schematic of dissected adult intestine showing major anatomical features overlaid with proposed transcriptional/functional domains shown in K. **(K)** Linear schematic representation of proposed transcriptional and functional domains of zebrafish intestine with potential regional gene markers and previously defined regional annotation. Boundaries should not necessarily be considered discrete, and domains may vary or overlap for different genes. Markers refer to proposed region-defining transcriptional markers determined using transgenic lines from this study (*) or from Wang et al. (^)[15]. Additional markers can be inferred from Fig 2 and S3 Fig, including markers for the most posterior region of the zebrafish intestine.

To determine if the transcriptional patterns and domains we detect in the larval zebrafish intestine also occur in adult stages, we assayed expression patterns in the adult (3+ months) zebrafish intestine using the same stable transgenic lines we queried in larvae. *Tg(rbp2a*:*GFP)* showed a similar expression pattern restricted to the anterior intestine as larval zebrafish; however, consistent with adult expression data (Fig 2B), high GFP expression did not extend to the most anterior IECs (Fig 5A–5B). This suggests that additional transcriptional domains or functional differences exist in the most anterior zebrafish intestine. We also found that *Tg(fabp6*:*GFP)* had a very similar pattern between larval and adult stages with a relatively small and discreet region of IEC GFP expression immediately after the second bend and between the segment 2 marker *TgBAC(lamp2-RFP)* (Fig 5F–5G) and the segment 1 marker *Tg(-4*.*5fabp2*:*DsRed)* expression domains (Fig 5H–5I). Collectively, this suggests that regional transcriptional programs in the zebrafish intestine are maintained between larval and adult stages. Further, the extent of functional homology between the zebrafish and mammalian intestine may be greater than previously appreciated (Fig 5J–5K). We propose a working model with at least 5 transcriptional/functional domains in zebrafish, although additional studies are needed to comprehensively resolve these domains, their interplay, boundaries, regulators, as well as the full nature and limitations of the homology between teleost and mammalian regional IEC programs (Fig 5K).

### Accessible conserved zCNEs in IECs are rarely IEC-specific and are likely open in most species and tissues

**Fig 6:**
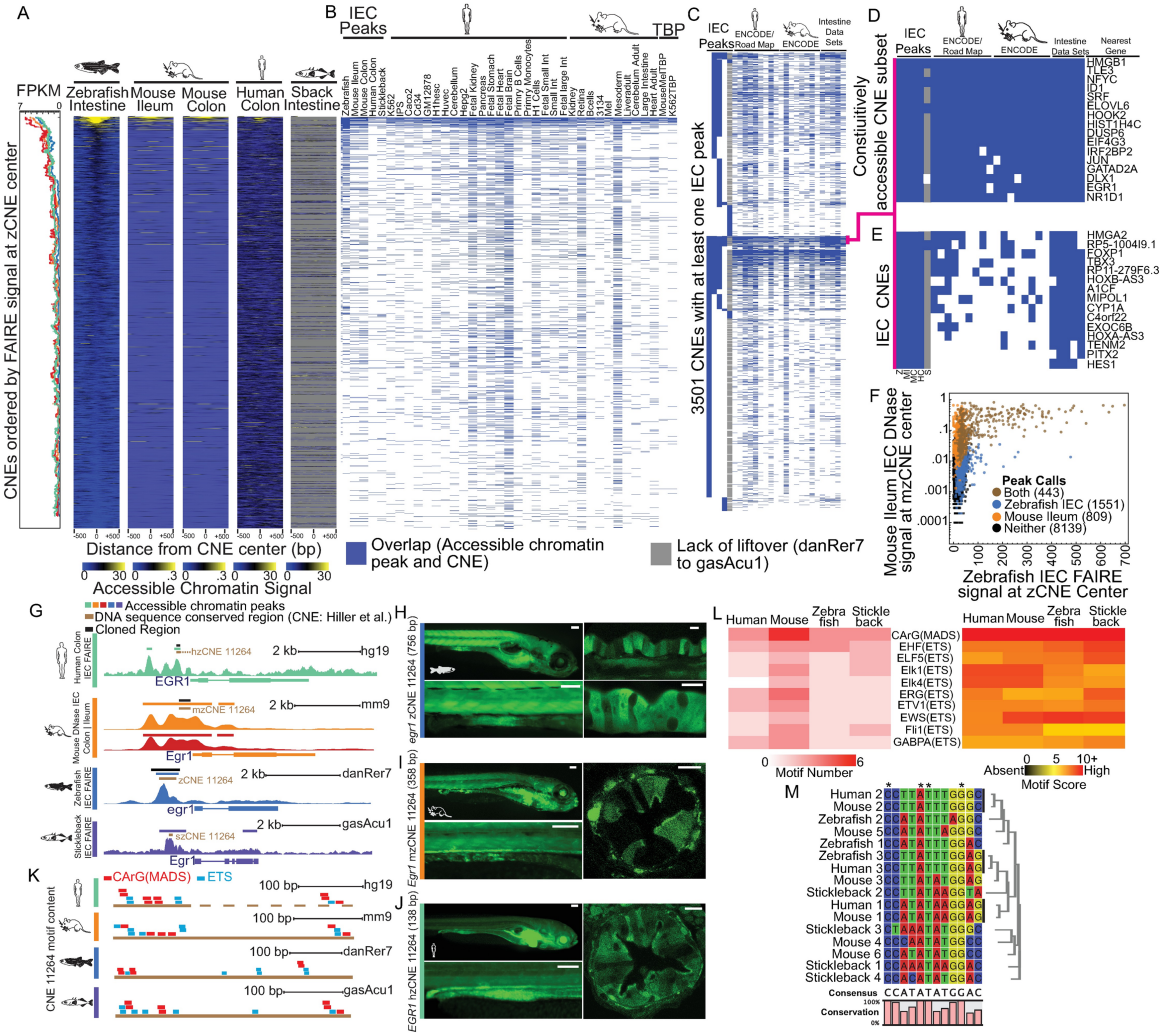
Intestinal epithelial cell (IEC)-specific accessible chromatin at conserved noncoding elements identifies IEC regulatory elements. **(A)** Conserved nonexonic elements (CNEs) ordered by zebrafish IEC Formaldehyde-Assisted Isolation of Regulatory Elements sequencing (FAIRE-seq) signal at zebrafish conserved nonexonic elements (zCNE) center. (Left) Moving median of Fragments Per Kilobase of transcript per Million mapped reads (FPKM) levels of CNEs nearest gene feature (250 gene window, 1 step; color scheme based on data sets presented in Fig 1A and throughout), showing the relationship between accessible chromatin level and transcription at these conserved regions. (Right) Heat maps of accessible chromatin signals at the 1,000 bp, surrounding the CNE center, highlighting the similarity of signal in IECs at CNEs in all 4 species. Gray represents no mappable signal or failure to liftover to the stickleback genome, as the stickleback genome was not included in the CNE set [55]. **(B)** Peak overlap of IEC accessible chromatin datasets and human and mouse ENCODE/Roadmap data sets with CNEs in each respective species. Overlap is defined as having at least 1 shared base pair. **(C)** Cluster analysis of CNEs with at least 1 IEC accessible peak overlap from any of the IEC datasets. **(D)** Subset of CNEs containing peak overlap from each of the zebrafish, mouse ileum, mouse colon, and human colon IEC datasets, as well as substantial overlap with additional mouse and human data from mostly unrelated tissues. **(E)** The entire group of CNEs that have overlap with zebrafish intestine, mouse ileum, mouse colon, and human colon but few other data sets, identify potentially highly conserved IEC-specific regulatory elements. **(F)** Scatterplot of accessible chromatin signal at the center of CNEs for zebrafish IECs and mouse ileum IECs, which shows a positive conserved relationship between accessible chromatin signal at these sites. **(G)** Accessible chromatin data from the *EGR1* locus highlighting the commonly accessible CNE_11264 in multiple species. **(H)** Whole-mount stereofluorescence of stable transgenic line harboring *egr1 Tg(zCNE_11264*:*GFP)* showing highest green fluorescent protein (GFP) expression in IECs in the mid intestine. (Bottom) Close-up view of zebrafish *egr1 Tg(zCNE_11264*:*GFP)* intestine. Scale bar 100 μm. (Right) Whole-mount confocal images confirming *egr1 Tg(zCNE_11264*:*GFP)* GFP expression in IECs. Scale bar 25 μm. **(I)** The same as H for stable line *Egr1 Tg(mzCNE_11264*:*GFP)*. **(J)** The same as H for stable line *EGR1 Tg(hzCNE_11264*:*GFP)*. **(K)** The distribution of CA/T-rich-G (CArG)/MCM1, AGAMOUS, DEFICIENS, and SRF (MADS box) and E26 transformation specific (ETS) motifs in human, mouse, zebrafish, and stickleback CNE_11264 show distinct motif distributions despite sequence conservation. Bronze bar represents conserved region. Dashed bar for human hzCNE_11264 represents the apparently conserved region that is adjacent to the hzCNE_11264 boundary marked with solid bronze bar. **(L)** Heatmap showing motif number (left) and highest motif score (right) for common motifs detected in CNE_11264. **(M)** Similarity relationship of CArG boxes detected in CNE_11264 shows the diversity of transcription factor binding sites (TFBS) despite overall sequence conservation. Numbering of CArG box sites is arbitrary.

In addition to TSS regions, we also specifically queried a published dataset of 54,533 zCNEs, of which 11,792 are also conserved to mouse and human, for accessibility in IECs [55]. Ordering zCNEs by the FAIRE-seq signal from zebrafish identified that the neighboring genes of the most accessible CNEs were also highly expressed in other-species IEC samples (Fig 6A). There was also a surprising overlap in the magnitude of accessibility at these conserved sites between species (Fig 6A, 6B and 6F). However, 48 of the 77 CNEs that were accessible in all zebrafish, mouse ileum and colon, and human colon IEC datasets were also accessible in at least 82% (14/17) of additional nonintestinal ENCODE and Human Roadmap datasets (Fig 6B–6D) [52–54]. This suggests that these regions are not specifically responsible for IEC expression. However, these frequently accessible conserved sites could represent a particular pan-vertebrate primitive transcriptional networks as zCNEs are commonly found at TSSs and near developmental and TF genes (Fig 6B–6E) [55].

Some of these conserved regions that are accessible in most tissues were near genes known to have roles in IEC biology, like *Egr1* [74], *Nr1d1* [75], and *Jun* [75], and we did not want to exclude that these regions could still be important in IEC expression. Cloning constitutively accessible regions from *nr1d1* and *jun* were negative for IEC expression. When we cloned the *egr1*-neighboring CNE regions from zebrafish (zCNE_11264), mouse (mzCNE_11264), and human (hzCNE_11264) and tested them separately using our reporter assay, unsurprisingly, multiple tissues showed GFP expression. However, we observed a distinct differential pattern across the intestine, with GFP expressing most highly in IECs in the mid intestine in zebrafish reporter lines representing all 3 species (Fig 6G–6J). TF motif searching identified multiple ETS and CA/T-rich-G (CArG)/MCM1, AGAMOUS, DEFICIENS, and SRF(MADS) box sites in all 3 species in CNE_11264, which are often immediately adjacent (Fig 6K), consistent with a serum response element that has been characterized at human *EGR1* [76–78].

We noticed the size of the conserved region and spacing and number of CArG and ETS binding sites of zCNE_11264 (458 bp) and mzCNE_11264 (352 bp) were greater than hzCNE_11264 (120 bp) (Fig 6G–6L). Searching for local CArG and ETS binding sites identified a neighboring cluster of additional CArG and ETS binding sites, approximately 200 bp outside of the conserved hzCNE_11264 region (Fig 6K). hzCNE_11264 was capable of driving similar IEC expression without these additional putative redundant conserved binding sites (Fig 6G, 6J and 6K). However, this highlights the imperfect nature of defining conserved regions across distantly related species and suggests that accessible chromatin maps combined with searching for common neighboring motif language seeds may identify additional sequence conservation information (S7J–S7L Fig). This same combination of ETS and CArG TFBS was found immediately upstream of *Egr1* in the stickleback genome, although zCNEs are not specifically annotated in stickleback (Fig 6K). Identifying these discrete nonoverlapping ETS and CArG binding sites at CNE_11264 in multiple species suggests that multiple ETS and CArG sites have functional relevance for this regulatory region (Fig 6K). However, this also demonstrates the complexity of identifying conservation that often relies on flexible, redundant regulatory logic (Fig 6K and 6L). Similarly, the diversity of detected CArG boxes also show how degenerate TF sequences can deviate while potentially maintaining a similar functional output (Fig 6M) [79].

### Accessible chromatin can identify conserved tissue-specific regulatory elements at broadly expressed genes

In an attempt to find zCNEs and upstream regulators that act primarily in IECs, we identified a group of 15 zCNEs that were not accessible in at least 9 out of 17 non-IEC tissues but were always accessible in IECs in zebrafish, mouse, and human (Fig 6E). Motif analysis of these IEC-accessible CNEs revealed common putative IEC-related TFBS in zebrafish, mouse, and humans such as GATA, HNF4A, HOX, CDX2, HNF6, HNF1, and TEAD (S7A–S7I and S8 Figs).

One of these zCNEs neighbors, the gene hairy and enhancer of split-1/hairy-related 6 (*HES1*/*her6*) (zCNE_44665), showed remarkable accessible chromatin specificity within intestinal datasets (Fig 6E; S9A and S9B Fig). Hes1 is a transcriptional repressor known to play diverse roles in many tissues including embryogenesis and neural and T-cell development [80–82]. Importantly, it also plays critical roles in the differentiation of IEC subtypes from intestinal stem cell progenitors and in mouse is expressed exclusively in the intestinal crypt [17,83]. While crypts are not present in the zebrafish intestine, and IEC progenitor or stem cells in fish have only been recently characterized [11], *hes1*/*her6* has been found to be expressed in a distinct subset of IECs in an analogous compartment at the base of zebrafish intestinal folds [84]. It is not fully known what genomic regions regulate *Hes1* IEC expression and if these regions control aspects of *Hes1*’s transcriptional response to microbes [85] or in intestinal cancer [86].

**Fig 7:**
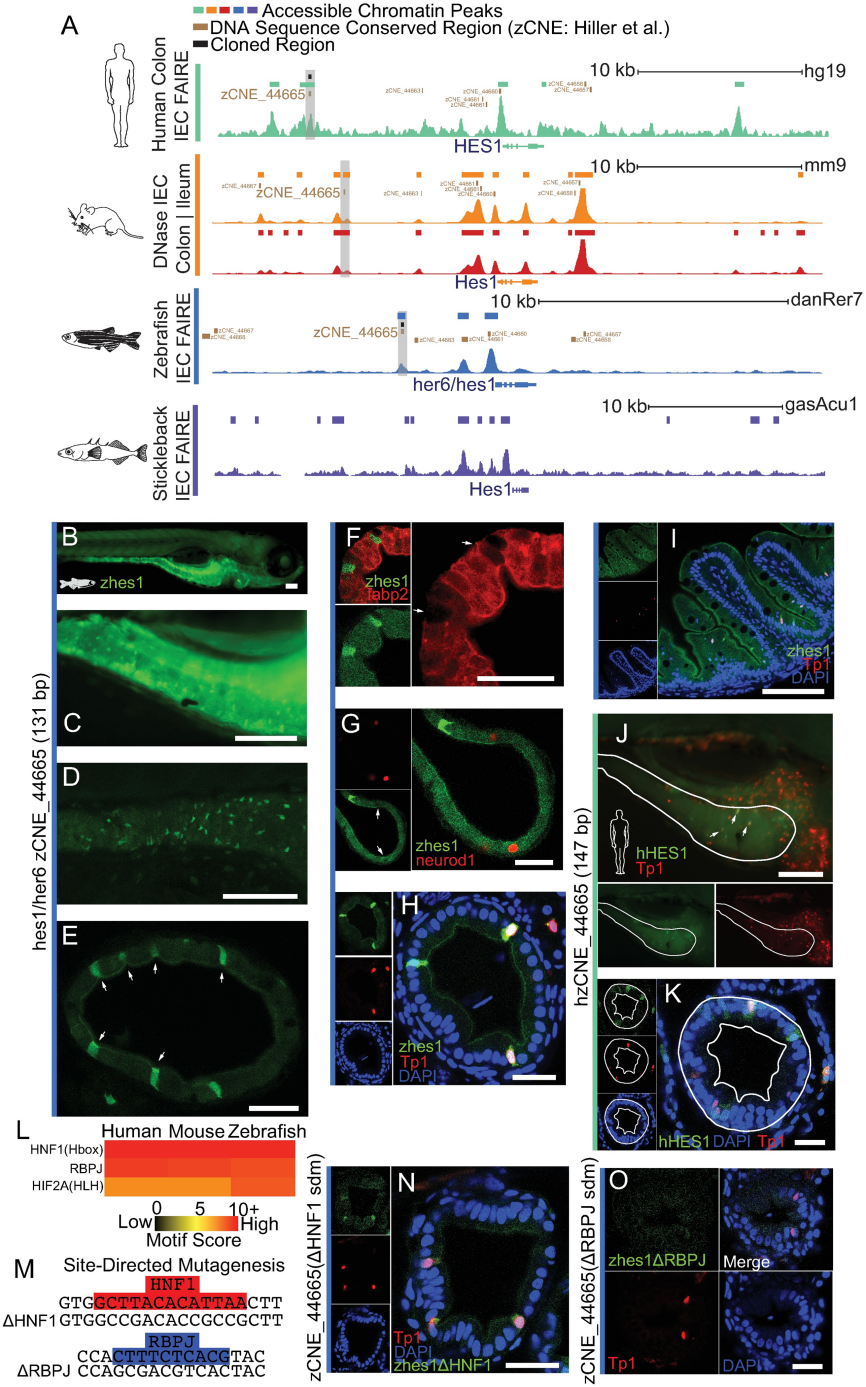
*Hes1* conserved nonexonic element (CNE)-driven expression overlaps with Notch signaling and marks a specific population of intestinal epithelial cells (IECs). **(A)** Accessible chromatin at *HES1* loci. (**B)** Whole-mount stereofluorescence of stable transgenic line 7 dpf zebrafish harboring *hes1 Tg(zCNE_44665*:*GFP)* showing green fluorescent protein (GFP) expression in IECs. Scale bar 100 μm. **(C)** Same as B, a closeup showing high GFP+ IECs in a population of cells. **(D)** Same as B, a confocal whole-mount z-stack maximum projection of zebrafish intestine showing high GFP+ in a population of IECs. **(E)** A confocal image of intestinal cross-section of 7 dpf *hes1 Tg(zCNE_44665*:*GFP)* showing GFP expression in IECs that appear at the base of slight invaginations (white arrows). Scale bar 25 μm. **(F)** A confocal intestinal cross-section of 7 dpf zebrafish shows lack of overlap between *hes1 Tg(zCNE_44665*:*GFP)* and enterocyte marker *Tg(-4*.*5fabp2*:*DsRed)*. GFP+ IECs lacking Discosoma sp. red fluorescent protein (DsRed) are marked with a white arrow. Scale bar 25 μm. **(G)** Lack of overlap between *hes1 Tg(zCNE_44665*:*GFP)* and the enteroendocrine marker *Tg(neurod1*:*TagRFP)* [87]. A reduction in background GFP can be seen at red fluorescent protein (RFP)+ cells (white arrow). Scale bar 25 μm. **H**) A confocal cross-section image shows overlap between hes1 *Tg(zCNE_44665*:*GFP)* and *Tg(EPV*.*Tp1-Ocu*.*Hbb2*:*hmgb1-mCherry)* in 7 dpf zebrafish. Individual channels are shown in inset with DAPI in blue. Scale bar 25 μm. **I**) A confocal image of cross-section of 8-week-old zebrafish intestinal folds in *hes1 Tg(zCNE_44665*:*GFP)* and *Tg(EPV*.*Tp1-Ocu*.*Hbb2*:*hmgb1-mCherry)*. **(J)** A stereoscopic image showing the overlap of whole-mount human *HES1 Tg(hzCNE_44665*:*GFP)* and *Tg(EPV*.*Tp1-Ocu*.*Hbb2*:*hmgb1-mCherry)* in a subset of IECs in 7 dpf zebrafish. Scale bar 100 μm. **(K)** A confocal cross-section of 7 dpf zebrafish showing the overlap of human *HES1 Tg(hzCNE_44665*:*GFP)* and *Tg(EPV*.*Tp1-Ocu*.*Hbb2*:*hmgb1-mCherry)*. Scale bar 25 μm. **(L)** Heatmap of common TFBS motifs in CNE_44665 in zebrafish, mouse, and human. **(M)** A schematic of site-directed mutagenesis on Hepatocyte nuclear factor 1 (HNF1) (top) and Recombination signal binding protein for immunoglobulin kappa J region (RBPJ) (bottom) putative binding sites from zCNE_44665. **(N)** A confocal whole-mount image showing the overlap between *hes1 Tg(zCNE_44665 ΔHNF1 sdm*:*GFP)* and *Tg(EPV*.*Tp1-Ocu*.*Hbb2*:*hmgb1-mCherry)*. Scale bar 25 μm. **O**) A confocal whole-mount image showing the overlap between *hes1 Tg(zCNE_44665 ΔRBPJ sdm*:*GFP)* and *Tg(EPV*.*Tp1-Ocu*.*Hbb2*:*hmgb1-mCherry)*. Scale bar 25 μm.

We were curious if our IEC chromatin data did discriminate important conserved regulatory regions that drove expression in IECs (Fig 7A). In our reporter assay zCNE_44665, a region approximately 3,600 bp upstream of the *hes1/ her6* TSS in zebrafish, revealed strong IEC expression and expression in other tissues including liver (Fig 7B–7D, S10 Fig). Whole-mount and cross-sections of 7 dpf fish identified that high GFP expression was within a subset of IECs, often at the base of slight invaginations of this cell layer (Fig 7B–7E, S10A Fig). These invaginations are ultimately analogous to the base of epithelial folds (rugae) seen in older fish and the intestinal crypt in mammals, although, at 7 dpf, substantial folds are not articulated (Fig 7D and 7E) [12]. This *Tg(zCNE_44665*:*GFP)* population did not overlap with cells that were positive with the enterocyte marker *Tg(-4*.*5fabp2*:*DsRed)* (Fig 7F and S10G Fig) [73], the enteroendocrine marker *Tg(neurod1*:*TagRFP)* (Fig 7G and S10H Fig) [87], or cells with characteristic goblet cell morphology. This suggests the *hes1*-neighboring conserved region zCNE_44665 drives GFP in an undercharacterized population of IECs that is distinct from these known differentiated IEC subtypes in zebrafish.

We scanned the sequence of the orthologous CNEs from zebrafish, mouse, and human and found TFBS for hepatocyte nuclear factor 1 (HNF1), hypoxia inducible factor 2 alpha (HIF2A), and recombination signal binding protein for immunoglobulin kappa J region (RBPJ) in all 3 CNEs (Fig 7L; S9A–S9C Fig). RBPJ is known to regulate *HES1* expression in the presence of Notch signaling [88], resulting in the alteration of the proportion of secretory and absorptive IEC lineages [17]. Therefore, we tested if this *Tg(zCNE_44665*:*GFP)* population was positive for Notch signaling. Crosses of *Tg(zCNE_44665*:*GFP)* with *Tg(EPV*.*Tp1-Ocu*.*Hbb2*:*hmgb1-mCherry)*, which uses a viral-derived promoter with Notch-responsive RBPJ binding sites [89], revealed substantial overlap between mCherry+ and GFP+ cells (Fig 7H and 7I, S10 Fig, S1 Movie). The mCherry+ IECs were always GFP+, and only 64.6% of GFP+ cells were mCherry+, which could be due in part to the relatively slow maturation of mCherry protein. This overlap suggests Notch signaling is important in regulating this element upstream of *HES1/her6* (Fig 7H and 7I, S10 Fig). A cross-section of dissected intestine from 8-week-old fish revealed a more sophisticated expression pattern in relationship to the now articulated intestinal folds (Fig 7I). GFP+ cells included cells at the base and typically in the bottom half of the intestinal folds and included cells positive for Notch signaling. Interestingly, these cells frequently had more apical nuclei than most IECs, whose nuclei typically are located basally within the epithelium (Fig 7I and S10E Fig).

To determine if a similar regulatory capacity was conserved to mammals, we then tested the hzCNE_44665 from human (approximately 10 kb from the TSS of *HES1*). This human region also drove intestinal expression that was similarly limited to a subset of IECs that often overlapped with Notch-positive cells (30.5% GFP+/mCherry+, 33.6% GFP+ only, 35.7% mCherry+ only) (Fig 7J and 7K). To determine if common putative TFBSs found in CNE_44665 were regulating expression in IECs, we generated a zebrafish line with the HNF1 binding site abolished through mutation in the zCNE_44665 GFP reporter construct *Tg(zCNE_44665 ΔHNF1*:*GFP)* (Fig 7L and 7M). We still identified GFP expression in a subset of cells that was coincident with Notch signaling in IECs from stable fish lines containing this construct (Fig 7N). However, when we abolished the RBPJ binding site, we found that high GFP expression was completely lost in IECs, including the subset of cells that overlapped with Notch-positive cells (Fig 7O). This suggests a conserved RBPJ binding site is necessary for expression in IECs and may contribute to common *HES1* regulation in fish and mammals in progenitor IEC populations (Fig 7O). Collectively, our chromatin data analysis is capable of distinguishing IEC regulatory regions and putative causative TFBS from complex regulatory landscapes that may regulate a broadly expressed gene’s transcription in IECs.

## Discussion

### Transcriptional similarities are detectable in IECs separated by 420 million years of evolution

Conserved regulatory elements that are stable over millions of years are likely to have analogous functions in their respective genomes and could coordinate conserved tissue-specific transcriptional patterns. However, identifying and functionally annotating these regions at individual loci or across a single genome is problematic, tedious, and does not accurately predict or robustly identify conserved function. As demonstrated above, our strategy of combining tissue-specific transcription and accessible chromatin datasets with conservation and TFBS prediction inferred from DNA sequences from multiple species identified putative conserved regulatory and functional information in IECs that could not have been identified by any one data set alone.

Tissues and cell types like IECs are defined by complex patterns of gene expression. Despite specializations in each animal species, IECs serve core inherent functions such as absorption and metabolism of dietary nutrients and xenobiotics and as a barrier to microbes residing within the intestinal lumen. As evidence for this common conserved function, we identified 470 orthologous genes expressed highly in IECs with relative tissue-specificity across 420 million years of vertebrate evolution. Functional conservation was maintained across a broad range of IEC biology, including genes involved in IEC subtypes, lipid transport and metabolism, and a response to microbes and inflammation. We highlight commonly expressed TFs because they may underlie the expression of conserved networks that are associated with IEC function, identity, and regionalization. *YBX1*, *HNF4A*, *ELF3*, *XBP1*, *ID3*, *HMGB2*, *IRF1*, *STAT3*, *GATA5*, and *OSR2*, amongst other TFs, appear to be more highly expressed in IECs than other tissues. Importantly, many of these highly expressed TFs also show enrichment for their cognate TFBS in accessible chromatin surrounding IEC signature genes in multiple species including the ETS factors *ELF3* and *ELF4*, *HNF4A*, *GATA5*, and *STAT3*.

### Complex regulatory mechanisms that specify regional function are conserved along the length of the zebrafish and mouse intestine

Our understanding of intestinal evolution has been hindered by a lack of information about the degree to which anteroposterior segments in extant vertebrate species are ancestral or derived traits. We found patterns of conserved expression along the length of the zebrafish and mouse intestine, suggesting that conserved discrete transcriptional regulatory programs may specify homologous duodenal, jejunal, ileal, and colonic segments along the zebrafish intestine [15]. We found striking evidence for transcriptional regulation underlying this conserved similarity, as the genomic region immediately upstream of the highly expressed IEC signature gene *FABP6*, in both zebrafish and mouse, was capable of driving GFP expression coincident between the zebrafish intestinal segment 1 and segment 2 domains [12–15]. We infer that this discrete segment specified by the expression of *FABP6* and other markers functions as the conserved homologous zebrafish ileum and that the zebrafish intestine is more completely defined as at least 5 distinct segments with further evidence that transcriptional domains similar to the duodenum, jejunum, and colon exist (Figs 2 and 5). Collectively, these results indicate that the transcriptional underpinnings of the well-characterized segmental program present in the mammalian intestine are ancestral to the last common ancestor with bony fishes and that the utility of the zebrafish as a model for human intestinal biology is even greater than previously appreciated.

### Accessible chromatin maps annotate regulatory regions in IECs in the absence of sequence conservation

Our initial analysis to utilize the presumed regulatory information at the TSS of IEC signature genes identified a number of regions with largely IEC-specific accessible chromatin status. However, we were unable to identify a substantial number of highly sequence-conserved regions at these TSSs. To circumvent the apparent lack of conserved regulatory information despite clear transcriptional similarities, we applied strategies through which regulatory information could be inferred without directly using traditional sequence conservation metrics. Searching for significantly enriched TF motifs found in accessible chromatin regions surrounding genes expressed in IECs in multiple species allowed us to identify common presumptive TF motifs that are used in the regulation of these genes, including *HNF1*, *HNF4A*, GATA, and ETS factors [60,62,63]. In addition, we looked for common predicted TFBS in TSS regions that appeared to have accessible chromatin in IECs but no strong sequence conservation. Accordingly, despite the lack of sequence conservation, the *RBP2(a)* promoter regions from zebrafish, mouse, and human are capable of driving highly similar expression in the IECs of zebrafish. This conserved expression is presumably largely due to common HNF4A and GATA motifs in zebrafish, mouse, and human that escape detection by commonly used sequence conservation metrics. Recently, a microbially responsive element in the zebrafish *angptl4* gene was shown to contain an element with HNF4A and GATA motifs that were involved in driving expression that is essentially identical to the expression pattern from our *rbp2a* fragment [68]. *Fabp2* also shows the same regional expression as *rbp2a* and *angptl4* in the intestine and has binding sites for HNF4A and GATA that are shared to mammals [72]. Furthermore, the TFs HNF4A and GATA, and FXR, that have putative binding sites in the regulatory regions from *Rbp2* and *Fabp6*, respectively, show intestinal expression patterns that appear to explain much of the regional IEC expression of these genes (S3G and S3H Fig). This suggests these common combinations [18] are conserved in regulating genes in the IECS of anterior intestines from teleosts to mammals. Intriguingly, HNF4A and FXR were also recently shown to mediate IEC responses to microbiota [26,90], indicating complex relationships between tissue-specific and microbially-responsive transcriptional programs in the intestinal epithelium.

### IEC-specific CNE at hes1/her6 drives expression in Notch-positive cells

We were able to identify a small number of highly conserved noncoding elements with apparent conserved IEC-specific chromatin accessibility, representing excellent candidates to understand conserved regulatory mechanisms that drive IEC expression (Fig 6 and S7 Fig). Our strategy highlights the utility of these data in identifying tissue-discriminating regulatory regions at genes that lack clear tissue-specific transcription and may be selectively regulated by discrete regulatory regions in different tissues. Similar strategies using available mammalian accessible chromatin datasets could annotate the remaining CNEs with putative functions. We focused on a CNE upstream of *HES1* because of its exceptional accessible chromatin specificity in the intestine (Fig 6E, S9A and S9B Fig) and the known importance of HES1 in IEC biology [91]. We identified a necessary RBPJ binding site within the *hes1* CNE that drove expression in IECs coincident with Notch signaling. Notch signaling likely plays a complex function at the *hes1* locus, as amongst the 6 *hes1/her6* neighboring zCNEs, 4 contain conserved RBPJ binding sites in their zebrafish, mouse, and human CNE counterpart (zCNE_44665, 44657, 44661, 44663), 1 CNE (zCNE_44658) contains an RBPJ site in zebrafish and human, and only 1 additional CNE (zCNE_44660) contains no RBPJ sites [92,93]. The only *HES1/her6*-neighboring zCNE containing a predicted binding site for HNF1 was zCNE 44665; however, loss of the HNF1 binding site was not sufficient to ablate expression in Notch-positive IECs, suggesting that other mechanisms confer the specific accessibility and expression in IECs. Due to the existence of other RBPJ sites at this locus, it seems unlikely that Notch signaling strictly underlies the IEC specificity, although tissue-specific expression of Notch ligands may also contribute to IEC expression [94].

Hes1 is expressed exclusively in the crypt of the mammalian intestine, including in stem cells and transit-amplifying progenitors coincident with Notch signaling [83,94,95]. Similarly, we see the zebrafish and human *HES1* CNE_44665 is capable of driving expression in IECs at the base of and bottom half of intestinal folds using our zebrafish GFP reporter assay, also coincident with Notch signaling [84]. It will be interesting to determine if Hes1 and the CNE_44665-marked cells play an analogous progenitor role in fish as well as mammals. Though we focused on the sequenced conserved region that also showed accessible chromatin specificity, a larger region outside of this conserved region showed intestinal accessible chromatin specificity in human and mouse (S9A and S9B Fig). Interestingly, a GFI1B binding site detected in the zebrafish zCNE, but absent in the mouse and human zCNE, was detected within the adjacent region that was accessible specifically in mouse and human intestinal datasets. GFI1B is a repressor TF that helps specify the IEC-subtype tuft cell [96]. This larger regulatory region may facilitate conserved regulation of *HES1* expression and restrict Hes1 expression in tuft cells, even though this GFI1B binding site is not found in a region that is conserved in sequence from zebrafish to human (S9A–S9C Fig) [96]. These results provide an important context for further exploring how conserved DNA regulatory regions and multiple TFs function cooperatively to regulate expression of the *HES1* gene in IECs and other distinct tissues.

### Towards a complete understanding of IEC specificity

While we framed this study around the idea that particular regulatory regions and transcripts could have specific functionality in IEC cell types, an interesting premise is that important regulatory regions and transcripts may be exclusive to multiple distinct organs or cell types, and these circuits could be conserved across species. Of course, even IECs are heterogeneous, so a complete understanding of IEC-specific programs will require higher resolution maps of IECs and multiple additional tissues [33]. Our examples of *Fabp6* and *Rbp2* do indeed seem to show high GFP expression that is largely limited to IECs (Fig 2). However, HNF4A and GATA also function in the liver [97] where *rbp2*a is expressed [66]; therefore, the definition of specificity and understanding of insulation of transcriptional regulation in different cell types requires further study. Though we believe our approach successfully identified genes and regulatory regions that are important in IECs, we did notice that the TSS at many IEC signature genes also showed accessibility in kidney and liver tissue (Fig 3E), suggesting an important overlapping utilization of the regulatory regions and gene functions in these other tissues. We do not want to minimize the importance of the concept that many regulatory regions are used in multiple cell types and should perhaps holistically be thought to function, be selected upon, and be conserved in an interorgan/cell-type regulatory network.

The strategies and methods we used here were able to detect diverse types of conserved transcriptional and regulatory information in fish and mammalian IECs; however, important limitations apply. Specifically, with our strategy, some pathways utilized broadly in many tissues, genes expressed in a small number of cells, or lowly expressed genes may be difficult to characterize for their conservation across species in the intestine and other tissues. For example, though the Wnt pathway functions in a broad range of tissues [98], it has been established as functioning in a similar manner in zebrafish and mammalian intestine. The Wnt coactivator TCF4 is tied to microbially regulated epithelial cell proliferation in zebrafish [99]. Remarkably, deletion of the Wnt pathway inhibitor, *Apc*, results in intestinal tumors in both zebrafish [100] and mammals [101]. However, only a small number of Wnt-related genes, including *FZD5*, were identified here as IEC signature genes, although individual ligands or pathway components may signal the use of broader pathways in IECs. As a result, the lack of highlighting a conserved gene or pathway cannot be considered as the lack of conservation, generally. Furthermore, the ability to find conserved function in a particular cell type, gene family, or pathway may depend highly on the proposed definition of conservation and be complicated by each genome’s unique history.

### The influence of orthology and teleost genome duplication on understanding conserved gene function and regulation

Teleosts underwent a genome duplication approximately 340 million years ago [102]. Approximately 20% of these duplicated gene pairs are maintained in the extant zebrafish and other teleost species, although the same duplicated genes are not always maintained in each genome [102–104]. Retained duplicated genes can undergo, amongst other fates, neo- and subfunctionalization specific to a lineage, spurring adaptation and potentially driving speciation [105]. This duplication can complicate comparative genomics and the parsing of function across vertebrate species with limited transcriptional profiling, but it also provides a rich platform for understanding gene evolution, function, and regulatory regions [106]. To create a focused strategy using genes that likely had maintained function and therefore detectable transcription and regulatory signal across species, our RNA analysis was largely limited to 1-to-1 orthologs across the 4 species we assayed, although our CNE analysis was essentially independent of orthology. We suspect that the diversity of types of regulatory information aren’t unique to genes with specific types of orthology across species, although novel regulation of gene function may more frequently arise during sub- and neofunctionalization of duplicated paralogs [105]. In addition, certain gene groups such as TFs are less likely to be lost following duplication in the zebrafish genome [102], suggesting that additional analysis may be required to uncover and integrate the functions and regulatory information that are contained within groups of genes that do not show 1-to-1 orthology.

### Traditional conservation metrics are not sufficient to identify all conserved regulatory information

We found instances at *Rbp2*, *Fabp6*, *Egr1*, and *Hes1* in which traditional sequence conservation metrics were not sufficient to fully identify common putative regulatory information from fish to mammals even when it is located approximately the same distance relative to the gene body in all species [57,107]. In all instances, accessible chromatin data provided additional context to identify conserved regulatory information. Importantly, because TFBS can be modular, conserved transcriptional regulation can occur with almost no sequence conservation signal being detectable across species. This property is largely due to the inherent nature of TFs because plasticity in the number, arrangement, and affinity of TFBS can result in nearly identical transcriptional responsiveness and output even in the absence of long stretches of sequence conservation. To more accurately identify conserved regulatory information, metrics are needed that incorporate short degenerate TF motifs or identify highly conserved short motifs between closely related species that are then identified as a conserved block in distantly related groups of similar species in syntenic regions. This information could be further anchored by accessible chromatin [108,109]. This may only partially circumvent the somewhat inherent statistical problem of short sequences arising by chance.

The interrogation of regulatory DNA sequences using assays such as GFP reporters can determine how complex orthologous regulatory sequences are interpreted in vivo. However, these assays are not necessarily sufficient to determine functional conservation across species. For example, orthologous TFs may show an altered TF binding motif preference in each species, although the regulation by this common factor is itself otherwise conserved. Alternatively, functional TFBS from one species may be invalidated by other neighboring sequences in cross-species assays, although the same TF is successfully regulating the same gene in each species. This issue is highlighted by the difference in AT% usage at the TSS of the 4 species used in this study that presumably is partially distinct from traditional TF binding content (Fig 3G). This property may partially be driven by more general sequence characteristics that require even less stringency in conserving specific long blocks of DNA like CpG methylation [64], nucleosome positioning, and overall dynamics of nucleosome accessibility [110], binding competition dynamics [111], or TSS selection [65] that may vary from organism to organism. Detecting sequences and principles that account for these confounding factors will need to be accomplished and compensated for before a complete understanding of the similarity and differences between analogous organs and transcriptional programs can be realized. Tissue-specific transspecies high-throughput enhancer activity assays [112] may be required to sufficiently sample and test transcription rules and conservation for specificity across species. These will need to be further combined with a similar deep understanding of posttranscriptional control and epigenetic mechanisms to develop a more complete picture of signals encoded in DNA and if they are commonly or divergently utilized.

### Prospectus

Our experimental strategy utilized IECs from healthy adult animals representing 4 vertebrate lineages to reveal conserved mechanisms underlying tissue- and region-specific IEC transcriptional regulation. These results provide an important frame of reference for future efforts to uncover similar mechanisms in IEC subtypes or in the context of other developmental stages, disease states, or environmental exposures. Many of the genes and upstream TFs broadly implicated here as conserved features of vertebrate IECs have already been implicated in human diseases (S1 Table) and in the intestinal response to microbiota [26,90], prompting further studies into the mechanistic relationships between transcriptional regulatory networks governing IEC identity, environmental sensitivity, and disease pathogenesis. We believe that using the strategy of simultaneously leveraging genome-wide data sets from multiple species can identify key ancient aspects of biology more quickly than studying any species alone.

## Materials and methods

### Ethics statement

Zebrafish studies were approved by the Institutional Animal Care and Use Committees of Duke University (protocol A165-13-06) and University of North Carolina at Chapel Hill (protocol 12–058.0). Stickleback studies were approved by the Institutional Animal Care and Use Committee of Stanford University (protocol 13834). Stickleback were collected under California Scientific Collecting Permit #3260. Studies involving human tissues were performed under University of North Carolina at Chapel Hill IRB approval numbers 10–0355 and 14–2445.

### Intestinal epithelial preparation and genomic assays for each vertebrate

#### Human FAIRE-seq and RNA-seq on adult human colon IECs

Total RNA was isolated from flash-frozen tissue samples (mucosal not whole tissue) from surgical colon resections distant from sites of disease and macroscopically normal as previously described [113] using the Qiagen RNeasy kit following the manufacturer’s protocol. DNA for FAIRE was isolated from the same samples as previously described [114]. Seven separate human colon IEC RNA-seq data sets were generated representing 7 individuals. Three separate, unrelated human colon IEC preparations were used to generate FAIRE-seq data sets from 3 individuals.

#### RNA isolation and RNA-seq analysis pipeline

Library preparation and mRNA sequencing were performed using protocols described previously [115]. Paired-end 50 bp mRNA reads were generated at UNC Chapel Hill (UNC-CH) High Throughput Sequencing Facility (HTSF) using the Illumina HiSeq 2000 platform. Each sample was aligned to a sex-specific hg19 genome using GSNAP [116], with a k-mer size of 15, 2 allowed mismatches per read, and RefSeq splice site annotations. In order to ensure high-quality alignments at regions containing genetic variability, we created a full-genome panel of SNPs with minor allele frequency greater than 0.05, obtained from the full phase 1, release 3 VCF annotation from 1,000 Genomes. We used this SNP panel in conjunction with the GSNAP -v option, which allows for SNP-tolerant alignments and improves mapping at variable sites. A post-alignment blacklist step was used to filter reads that were aligned to problematic, highly artifactual regions identified by ENCODE. Quantification of RPKM values was conducted using an in-house script with RefSeq gene annotations.

#### FAIRE and FAIRE-seq analysis pipeline

FAIRE was performed as described previously [114]. Using the Illumina HiSeq 2000 platform, 50 bp single-end sequences were generated at UNC-CH HTSF. Reads were filtered requiring a quality score of 20 or greater in at least 90 percent of nucleotides, and adapter contaminated reads were removed with TagDust [117]. Additionally, no more than 5 reads with identical sequence were retained. Nonfiltered reads were aligned with the GSNAP software [116] to sex-specific hg19 genomes using k-mer size of 15 and allowing 1 mismatch per read. In order to ensure high-quality alignments at regions containing genetic variability, we used the GSNAP -v option with SNP annotation derived from 1,000 Genomes, as described in RNA alignment section above.

### Mouse

Mouse tissue dissection and IEC extraction protocols and all initially processed data were previously described (GSE57919) [16].

### Zebrafish

#### FAIRE-seq and RNA-seq on adult zebrafish IECs

Accessible chromatin and transcriptome data from zIECs was generated from wild type TL strains reared in the Zebrafish Aquaculture Core Facility at UNC-CH. A total of 3 replicates for RNA-seq and 4 replicates for FAIRE-seq were generated, each replicate from 3 pooled intestines of isolated adult IECs. Conventionally raised adult fish were fed twice daily with Great Salt Lake strain brine shrimp (*Artemia*, Aquafauna Bio-Marine, ABM-GSL-TIN-90) supplemented with flake food (5 parts Tetramin Flakes Aquatic Ecosystems, 16623; 1.5 parts Zeigler Aquatox Flakes, Aquatic Ecosystems, AX5; 1.5 parts Spirulina Flakes Aquatic Ecosystems, ZSF5; 1 part Cyclop-eeze Argent Chemical Laboratories, F-CYCL-FD30-CS; 1 part San Francisco Bay freeze-dried brine shrimp Aquatic Ecosystems, SB113).

To isolate zebrafish IECs, intestines were dissected, splayed, and washed extensively with ice-cold 1x PBS with care taken to remove as much intestine-associated fascia, adipocytes, and blood vessels as possible. For each of the 3 RNA-seq replicates, 3 washed intestines were transferred into dissociation reagent 1 (DR1; 30 mM EDTA, 1.5 mM DTT, 0.5x Complete protease inhibitors [Roche], in 1x PBS) for 15 minutes on ice. Segments were transferred to Dissociation Reagent 2 (DR2; 30 mM EDTA, 0.5x Complete protease inhibitors [Roche], in PBS) and moderately shaken by hand for 5 minutes until most epithelial cells were isolated in the suspension. Intestinal lamina propria was removed, and 8 ml of cold 1x PBS was added to the cells on ice. Cells were pelleted at 500 x G at 4°C, washed once with 13 ml of cold 1X PBS, and resuspended in 0.5 ml cold 1x PBS. A 0.4 ml fraction was used for FAIRE, and a 0.1 ml fraction was reserved for RNA extraction.

FAIRE-seq was performed as described [114] with minor modifications. Briefly, freshly isolated IECs were directly fixed for 5–10 minutes in 10 ml of 1%–3% w/v formaldehyde solution (in 1x PBS) at room temperature and gentle rocking. Glycine (2.5 M) was added to a final concentration of 125 mM to quench the formaldehyde. Cells were pelleted at 600 x G and washed 3 times in cold 1x PBS without dissociating the pellet. Fixed and washed cell pellets were flash frozen and stored at −80°C. Cells were lysed in 2 ml Lysis Buffer A (10 mMTris-HCl [pH8.0], 2% [vol/vol] Triton X-100, 1% SDS, 100 mM NaCl and 1 mM EDTA) and sonicated using a Branson Sonifier 450D equipped with a microtip for 6–13 cycles (1 second burst, 0.5 second pause, for 30 seconds/cycle at 70% intensity) allowing samples to cool on ice for 1 minute between cycles.

FAIRE-seq libraries were prepared using the TruSeq kit (15025064, Illumina) according to manufacturer’s specifications with the following exceptions. One hundred nanograms of input FAIRE DNA was used for all zIEC samples. Adaptors were diluted 1/10 prior to ligation. Libraries were verified using an Agilent Bioanalyzer by the UNC-CH Bioinformatics and Genomics Core facility and sequenced (2 libraries multiplexed per lane) using Illumina HiSeq 2000 at the UNC-CH HTSF. FAIRE-seq sequencing results were processed and mapped to the zebrafish genome (danRer7/Zv9) using Bowtie.

Total RNA was extracted from adult zebrafish IECs using TRIzol Reagent (Invitrogen). Two micrograms (in 50 μl RNase-free water) were used for TruSeq library preparation (performed by the UNC-CH HTSF) for mRNA Illumina sequencing using 2 x 50 bp paired-end reads. Four samples were multiplexed per lane. Reads were mapped to danRer7/Zv9 using TopHat v1.4.0. Normalized Fragments Per Kilobase of transcript per Million mapped reads (FPKM) expression values were generated using cufflinks v2.0.2 with default parameters and gene annotations from Ensembl Zv9 release 71.

### Stickleback

#### FAIRE-seq and RNA-seq on adult stickleback IECs

Wild-caught adult stickleback fish (2 female, 1 male) from Friant River, California (Approximate GPS coordinates: N36:58:47-W119:43:51) were used in this study (CA Scientific Collecting Permit #3260). A total of 3 replicates for RNA-seq and 1 replicate for FAIRE-seq were generated from isolated adult IECs. IECs were isolated from the anterior intestinal tract starting just posterior to the pyloric sphincter below the stomach, with the rectum removed (distal-most portion, approximately 5 mm) [118]. RNA was isolated, and FAIRE performed exactly as described for the zebrafish IEC samples. FAIRE-seq reads generated on an Illumina HiSeq 2000 by Duke Sequencing and Genomic Technologies Shared Resource were aligned to the stickleback reference genome (gasAcu1) using Bowtie. Two micrograms of total RNA were used for TruSeq library preparation for sequencing 50 bp paired-end reads. Reads from Stickleback Adult IECs were mapped to gasAcu1 using TopHat2 (v2.0.9) with default parameters. Normalized FPKM expression values were generated using cufflinks (v2.1.1) [119] with gene annotations from Ensembl BROAD S1/gasAcu1 release 76.

### RNA levels and downstream analysis

#### Orthology definitions

Orthology definitions were extracted from Ensembl Biomart (December 2014; Ensembl Genes 78) by identifying orthologs using a compiled list of “ortholog_onetoone” homology types for each species with orthology to zebrafish as a common comparison. Ensembl biomart, unique identifiers, and orthology definitions were used to compare genes across species when necessary. Ensembl orthology calls were used without manual curation, which may have resulted in the inclusion of rare false positives or false negatives. For example, zebrafish *rbp2a* was included as a 1-to-1 ortholog by Ensembl despite the existence of a rapidly evolving paralog *rbp2b* [120].

#### IEC signature genes

To identify intestinal epithelial signature genes, we combined our RNA-seq data with RNA-seq data from multiple mouse tissues using 4,248 1-to-1-to-1-to-1 orthologs with detectable mRNA signal across tissues [29]. We calculated a pairwise distance matrix using Pearson correlation based on all expressed genes to estimate the similarity of all samples. We performed PCA and found that PC1 separated intestine samples from all other tissues, and we extracted the genes correlating with PC1 using a PC loading correlation threshold of >0.7. We removed ribosomal proteins from downstream analyses. R studio (https://www.rstudio.com/) was used to run custom R scripts to perform PCA (FactoMineR package), hierarchical clustering (stats package), and to construct heatmaps, scatter plots, and dendrograms. Complete linkage clustering was performed with hclust using correlation distance metrics through dist. Packages ggplot2 and gplots were used to generate data plots.

#### DAVID analysis

GO term and KEGG Pathway analysis were performed using DAVID v6.7 with Human Ensembl Gene IDs as input (http://david.abcc.ncifcrf.gov) [121].

#### EnrichR

Mouse and Human Gene Atlas tissue enrichment overlap was performed using Enrichr with Human Official Gene Symbols as input (http://amp.pharm.mssm.edu/Enrichr/) [122].

#### Processing of intestinal regionalization data

Normalized single-channel intensities were z-scored separately for each dataset corresponding to whole duodenum, jejunum, ileum, and proximal colon from WT adult conventionally-raised mice (GSM434935) [47] and 7 sections of equal length of WT adult conventionally raised whole zebrafish intestine (GSE20884) [15]. Zebrafish 1-to-1 orthologs (zebrafish to mouse) with at least 1 intestine section’s z-score value greater than 0 were clustered using a complete clustering method with an uncentered similarity metric in Cluster 3.0 [123] to identify genes that show differential expression levels across the zebrafish intestine. Consistent with previously published data, a cluster corresponding to genes with relative mRNA levels highest in the first 5 sections and an additional cluster in the last 2 sections were apparent [15]. In addition, an uncharacterized cluster of genes with high mRNA levels only in section 5 were found. For genes within these 3 clusters, linear correlation coefficients were generated between values for the 7 zebrafish regional segments and 7 mouse regional segment data sets (3 of which were generated by linear interpolation between the 4 [duodenum, jejunum, ileum, and colon] adjacent mouse segments, effectively generating expression values for 7 total mice segments). The data set was then filtered to exclude 1-to-1 orthologs with a linear correlation coefficient below .6 when comparing zebrafish and mouse relative expression values. This identified mouse genes that had similar expression patterns along the length of the intestine as their orthologs in zebrafish. For visualization, z-scored section values were median centered for each gene and then sorted by each mouse intestinal segment to identify the genes that showed the highest relative level of mRNA for each particular segment in mouse, which frequently corresponded to similarly regional intestinal expression in zebrafish. Genes were then separated by their original zebrafish cluster to effectively show which genes had similar patterns relative to their mammalian counterparts and anatomy. The genes in the cluster showing high relative expression in the anterior sections of the zebrafish intestine were sorted both by mouse duodenum and jejunum values (Fig 2, S3 Fig).

### Accessible chromatin and downstream analysis

#### Peak calls

Peak calls on accessible chromatin data were generated using MACS2 [124] with default conditions except with broad region calling off and using the reported q-value cutoff for each species (S3 Table). Peaks were merged for each replicate, collapsing overlapping peaks to create a final list of peaks used for analysis. Peak calls for ENCODE and Human Roadmap samples were downloaded directly using previously published data (https://www.encodeproject.org/) (S3 Table). In all cases, overlaps were called if 1 base pair was shared between different features. Additional routine genomics data processing and visualizations were performed using a locally installed Galaxy instance (13771:7a4d321c0e38) including the Java-genomics-toolkit (https://github.com/timpalpant/java-genomics-toolkit) and the main Galaxy server (https://usegalaxy.org).

#### TSS definition

TSS and TTS gene coordinates were downloaded using the UCSC table browser. For hg19 and mm9, Refseq definitions were used because they provide predominant gene isoform coordinates, allowing for the use of the most characterized and utilized TSS. Due to lack of Refseq annotation of many predicted genes in danRer7 and gasAcu1, TSS and TTS coordinates were extracted using the UCSC table browser from the Ensembl database. When multiple gene isoforms were present, a representative was chosen at random. Ensembl biomart and orthology definitions were used to compare genes across species when necessary.

#### Gene regulatory domain definition

Maximum and minimum gene TSS and TTS coordinates from Refseq and Ensembl genes, extracted as explained in **TSS definition** above, were utilized for each gene to include all DNA within the largest possible transcriptional region. To generate local regulatory domains, 10 kb were added to both the beginning and end of this region to increase the region associated with each gene by a total of 20 kb.

#### TFBS motif enrichment and annotation

Motif searching was performed on genomic sequence using the “homer2 find” (http://homer.salk.edu/homer/index.html;v4.7.2) [59] command with the motif library provided with repetitive, yeast, and plants motifs excluded. Results for common short degenerate motifs that were consistently found in the majority of motif-annotated sequences that did not rely on statistical enrichment were excluded from presentation. Motif enrichment was performed on repeat-masked genomic sequence using the findmotifs.pl script from homer2 and the full homer2 motif library (http://homer.ucsd.edu/homer/motif/HomerMotifDB/homerResults.html). When appropriate, background sequences were used as noted or using the corresponding sequences associated with the bottom 1,000 genes with the lowest PC1 correlations.

### Conservation metrics

#### zCNE definitions

zCNE coordinates (danRer7) [55] and corresponding coordinates from mouse zCNEs (mm9; mzCNEs) and human zCNEs (hg19; hzCNEs) were provided by the lab of Dr. Gill Berjerano (http://bejerano.stanford.edu/zebrafish/public/html/). Liftover of zCNEs from zebrafish (danRer7) to stickleback (gasAcu1) was performed using the LiftOver utility from UCSC (https://genome.ucsc.edu/cgi-bin/hgLiftOver).

#### LiftOver

The UCSC LiftOver tool was used to liftover regions (e.g. TSS-1,000 bp excluding any coding regions) from the zebrafish (danRer7) genome to mouse (mm9) and human genomes (hg19). Liftover to either mammalian genome was considered as conservation for a particular gene.

#### MAF blocks

MAF blocks were extracted from 100-way multiZ new (hg19) to hg19 and mm10 using zebrafish TSS-1,000 bp regions, excluding any coding regions for IEC signature genes. Genes that had MAF blocks of at least 10 bp from zebrafish to mouse or human were considered as conservation for a particular gene.

### Cloning and generation of reporter constructs

Putative regulatory elements were amplified from genomic DNA with primers containing FseI and AscI restriction site overhangs and then cloned, maintaining orientation relative to their native TSS, into the p5E-FSE-ASC entry plasmid (381) (http://tol2kit.genetics.utah.edu/; *Tol2kit v1*.*2*) [125]. Putative clones were confirmed by PCR and sequencing to ensure an exact match to genomic sequence from the corresponding species. Four-way LR reactions were performed using the LR Clonase II Plus Kit (12538120, Invitrogen) combining p5E-FSE-ASC modified with a putative regulatory element, pME *cFos* (S4 Table), p3E EGFP (366), and pDestTol2pA2 (394) using the provided protocol. This generated a single plasmid recombined with a putative regulatory region upstream of a minimal mouse *cFos* promoter-driving eGFP and flanked by Tol2 transposon insertion sites, which was confirmed to contain the putative regulatory region by PCR. Site-directed mutagenesis constructs were generated by randomly changing key bases over 10 bp in TFBS, detected by Homer within the zebrafish *hes1*/*her6*-neighboring zCNE_44665. Homer could no longer detect the targeted TFBS using the mutated genomic sequence in the context of the otherwise original zCNE_44665 sequence. For each site-directed mutation, complementary primers containing the 10 bp mutated region flanked by wildtype 20 bp sequences on either side were used in circular PCR with the original zCNE_44665 containing 381 plasmid as template followed by DpnI treatment to digest methylated plasmid. Site-directed mutagenesis was confirmed by sequencing.

#### In vivo reporter assay

In vivo reporter assays were conducted at Duke University with zebrafish reared using established methods [126,127]. The following existing lines were used in this study: EK, *TgBAC(lamp2*:*RFP)*^*pd1044*^ [71], *Tg(-4*.*5fabp2*:*DsRed)*^*pd1000*^ [73], *Tg(neurod1*:*TagRFP)*^*w69*^ [87], and *Tg(EPV*.*Tp1-Ocu*.*Hbb2*:*hmgb1-mCherry)*^*jh11*^ [89]. Note that *TgBAC(lamp2*:*RFP)*^*pd1044*^ is distinct from *TgBAC(lamp2*:*RFP*)^*pd1117*^ [71] but was generated using identical methods. New transgenic alleles and lines generated in this study are listed in S5 Table. Constructs were injected using a Picospritzer into EK zebrafish embryos at the 1 cell stage using an injection cocktail including 150 ng of the Tol2 enhancer plasmid, 10% (V/V) of Phenol red solution (Sigma; 0.5% in DBPS), 200 μM HEPES Buffer pH 7.0 (CellGro), and 250 ng transposase mRNA and water to a total of 5 μl using approximately 2 nl or approximately 70 pg per embryo as described [58]. Typically, mosaic GFP IEC expression could be scored at the F0 generation following injection. F1 lines were further screened for GFP expression at 1 dpf to identify transgenics and for IEC expression through 7 dpf. For each construct, at least 2 independently generated F1 lines were identified as having consistent expression patterns in IECs, and, typically, 1 of these lines was used as a representative for imaging and analysis. A construct was considered negative for driving IEC expression if no mosaic IEC expression was detected at the F0 generation, and the F1 population generated multiple GFP positive offspring from distinct crosses that subsequently failed to show clear IEC expression at 7 dpf. For constructs with negative results for IEC expression in larvae, adult tissue was not assayed.

#### Imaging

Whole-mount images profiling GFP expression in the zebrafish were taken on a Leica M205 FA stereofluorescence microscope except for images with maximum or average projections, which were taken on a Leica SP8 confocal microscope. For consistency, Figs 4A, 5I, 5J and 6B whole-mount images were reflected on the vertical axis to commonly orient fish across all figures. Two-hundred-micrometer thick cross-section images were generated with a Vibratome (Leica) and visualized on a Leica SP8 confocal microscope using mounting media with DAPI (Vectashield H-1200).

## Supporting information

S1 Fig Relationship between relative mRNA levels for IEC data sets from 4 different species. **A**) Pairwise scatter plots showing FPKM values for IEC datasets from four species for IEC signature genes (red) and non-IEC signature genes (black). **B**) Heatmap of linear correlation coefficients for pairwise comparison between IEC datasets and mouse non-IEC datasets [29] for IEC signature genes, non-IEC signature genes, and transcription factors. **C**) Scatter plots of IEC FPKM values for IEC signature (green) and non-IEC signature (black) transcription factors. **D**) Heatmap of FPKM values or IEC and other tissues for representative genes that are specific to IEC subtypes. Despite the lack of Paneth cells in zebrafish, XBP1, a Paneth cell associated transcript, is highly expressed in zebrafish IECs. **E**) Heat map of expression levels for IECs and other non-IEC tissues [29] ordered by PC1 correlations from Fig 1E. Black vertical bar marks IEC signature genes.
(PDF)

S2 Fig Comparison of replicates for RNA-seq and accessible chromatin data sets. **A**) Heatmap of clustergram using complete linkage with a correlation uncentered similarity metric for arrays using log10 FPKM values from IEC datasets for all species for 1to1to1to1 orthologs. Genes are ordered by average log10 FPKM values across all replicates. **B**) Heatmap of clustergram using complete linkage with a correlation uncentered similarity metric for arrays and genes using log10 FPKM values for all genes from stickleback IECs. **C**) Same as B for zebrafish IECs **D**) Same as B for human IECs. **E**) Heatmap of clustergram using complete linkage with a correlation uncentered similarity metric for arrays and genes using the sum of accessible chromatin signal (sequencing counts) at the TSS+/-50 bp (the 100 bp window centered on the TSS) for 1-1-1-1 orthologs. Arrays are median centered and normalized using Cluster 3.0. **F**) The same as E for zebrafish IECs. Stickleback data is also provided and ordered by the zebrafish clustering. **G**) The same as E for human IECs.
(PDF)

S3 Fig Conserved regional transcriptional similarities along the intestine in zebrafish and mouse. **A**) Consistent with a previously published result [15], a heatmap of a cluster analysis of previously published single channel intensity z-scored mircroarray mRNA levels from adult zebrafish whole intestine dissected into 7 equal length segments. Cluster analysis includes zebrafish 1to1 orthologs (zebrafish to mouse) with at least one intestine section’s z-score value greater than 0 clustered using a complete clustering method with an uncentered similarity metric in Cluster 3.0. **B**) Same as A for adult mouse whole intestine sections from using previously published mouse data [47]. **C**) Heatmap of 1to1 orthologs from the zebrafish cluster in (A) marked by a blue bar for genes with a linear correlation over 0.6 between the 7 zebrafish sections and 7 values generated by linear interpolation between the z-scores for the 4 mouse segments sorted by z-score in mouse jejunum. Evidence for conserved transcriptional regulation for genes most highly expressed in zebrafish sections 4–5 and mouse jejunum suggest nuanced expression patterns are conserved. **D**) Same as C for the orange cluster highlighting an apparent ileum signature. Additional zebrafish genes of potential interest due to their expression patterns, but without 1to1 orthology, are added by hand. **E-F**) Same as (C) for the green cluster broken into two groups of genes that are lowly (E) or highly (F) relatively expressed preferentially in zebrafish sections 6–7 sorted by values from the mouse colon. Single channel z-score values across intestinal segments in (**G**) zebrafish and (**H**) mouse for transcription factors that have binding sites in the promoters of Rbp2 and Fabp6 show expression patterns that may help specify regional intestinal expression patterns in IECs across species. Colored dots on y-axes correspond to scales for colored data sets. Numerical values can be found in S1 Table.
(PDF)

S4 Fig Consistent cross-species relationship between expression and accessible chromatin suggests information in promoter regions may help regulate expression specificity in IECs. **A**) Accessible chromatin signal at 1000 bp surrounding the TSS of 1to1to1to1 orthologs ordered by PC1 correlation used to identify IEC signature genes for zebrafish, stickleback, mouse ileum, mouse colon and human colon accessible chromatin data (Right). Moving median (Left) for FPKM of associated genes (250 gene window, 1 step; color scheme used throughout and shown in B) IEC signature genes are marked by a black vertical bar. **B**) Moving median (250 gene window, 1 step) for accessible chromatin signal at TSS from IECs based on ordering in A). Numerical values can be found in S1 Table. **C**) Heatmap of common motif enrichment within the TSS-1000 bp region for IEC signature genes. **D**) Heatmap of common motif enrichment within IEC accessible chromatin peaks within the region TSS-1000 bp for IEC signature genes.
(PDF)

S5 Fig Adult zebrafish express transcriptional domains that are positionally similar to those seen in larvae. **A**) Accessible chromatin signal for RBP2(a) human, mouse, zebrafish and stickleback loci. Accessible chromatin peaks colored based on color scheme used throughout for IEC samples, conservation from zebrafish to human or mouse; bronze, and cloned region; black). **B**) Accessible chromatin signal for FABP6 loci from human, mouse, zebrafish and stickleback. Accessible chromatin peaks colored based on color scheme used throughout for IEC samples, conservation from zebrafish to human or mouse; bronze, and cloned regions; black). **C**) UCSC screenshot for Phylop conservation score and Multiz hg19 alignment (top) and Phylop conservation score for Multiz mm9 alignment (bottom) for representative species at the region immediately upstream of *FABP6/Fabp6* transcription start site and highlighted in bronze in B. Highlighted are predicted TATA-box motifs that overlap the conserved region in both mouse and human. While a conserved signal is detected from human and mouse to zebrafish only the TATA-box appears to be conserved.
(PDF)

S6 Fig Cloning strategy, cFos injection control, and autofluoresence background. **A**) Simplified schematic of 4-way LR recombination cloning. Briefly, fragments amplified from a genomic template with primers containing FseI and AscI overhangs are cloned into a 5’ entry vector. A 4-way LR reaction combines the 5’ entry containing the putative regulatory element with a middle entry vector containing the minimal cFos promoter (S4 Table) and 3’ entry vector with eGFP into a destination vector containing Tol2 insertion sites [125]. **B**) A cFos control vector was generated to test for potential insertion background expression and the expression capacity of the cFos minimal promoter in IECs as this construct presumably can act as an enhancer trap when inserted into the genome without additional regulatory information. Comprehensive testing of potential insertion influence with this control vector is difficult, however typically no or little expression in IECs was found and none of the cFos control expression fish drove expression patterns that were consistent with the elements tested in the main body of the paper (**C-F**). While wholemount stereoscopic microscopy of fish containing this control construct did not readily detect IEC GFP expression that we found when testing putative IEC regulatory elements (Figs 4–6). We also performed confocal microscopy on a subset of fish as this is typically more sensitive at identifying localized GFP signal. We note below where limited IEC expression existed in the control lines we profiled, however any IEC expression was not usual and not consistent between control lines. **C**) Developmental pattern of GFP expression of cFos control line 3m. **D**) Developmental pattern of GFP expression of cFos control F1 line 7m **D’**) Cross-section of cFos control line 7m shows light expression in a goblet cell. **E**) Developmental pattern of GFP expression of cFos control F1 line 9m. **E’**) Cross-section of cFos control line 9m shows no detectable GFP expression in IECs. **F**) Developmental pattern of GFP expression of cFos control F1 line 9m. **F’**) Cross-section of cFos control line 9m shows light GFP expression in IECs. Figures (C-F) are presented on a black background for clarity. Different fish from each line can be represented on different days. Lines 3m shows yolk autofluorescence near the intestine consistent with developmental differences that can disappear variably in developing fish due to environment or genetic background. **G-I**) Stereomicroscopy images of 7dpf wild-type EK fish without any GFP insertions show the typical autofluoresence signal that can be detected within the intestinal lumen. For each image the number of milliseconds of exposure for the GFP signal is listed. Scale bar 100 μm.
(PDF)

S7 Fig Motif content for CNEs with IEC chromatin accessibility. **A**) CNEs showing accessible chromatin largely in IECs corresponding to Fig 6C and 6E. Common results of Homer transcription factor binding site motif search in all three species for CNEs **B**) PITX2 CNE_12472, **C**) MIPOL1 CNE_18098, **D**) TENM2 CNE_11789, **E**) C4ORF22 CNE_41249, **F**) A1CF CNE_6073, **G**) HMGA2 CNE_38779, **H**) HOX3_AS3 CNE_23732, and **I**) RP11-279F6.3/KIF23 CNE_21883. Not pictured are CNEs that had no motifs in common in all three species. Corresponding CNE size [55] between **J**) zebrafish and mouse, **K**) zebrafish and human, and **L**) mouse and humans reveals that linked CNE size is often smaller when comparing from the zebrafish anchor genome to alignment genomes suggesting conserved regulatory information may be lost using conservation strategies. Dashed black line represents a slope of 1. In a few cases CNEs from zebrafish can map to non-continuous regions of the alignment genomes. In this case, the cumulative size of these regions is summed to generate a single size for each corresponding CNE in each genome.
(PDF)

S8 Fig Cluster-based strategy to identify potential CNEs that are largely accessible in IECs. **A**) Heatmap of cluster analysis of overlaps between CNE regions and accessible chromatin peak calls for IECs and other tissues. Clusters showing CNEs with relatively specific accessible chromatin in IECs are marked as A1 (red), A2 (orange), and A3 (yellow). CNEs that appear largely constitutively accessible in IEC and other tissues in all species are labeled Constitutive (green). **B**) Blow up of A1 cluster showing CNE names [55] and nearest genes. CNEs marked with asterisks are used in motif analysis in E. **C**) Same as B for A2 cluster. **D**) Same as B for A3 cluster. **E**) Common motifs found in CNEs identified in A-D for a subset of CNEs near genes of interest based on known IEC biology or due to the specificity of accessible chromatin to IECs in multiple species. **F**) Motif enrichment to identify motifs that are more often found in CNEs that show chromatin accessibility in IECs reveals multiple TFBSs important in IEC biology like HNF1, CDX2, and HNF4A. Various groups of non-IEC specific CNEs are used as backgrounds for motif enrichment as labeled. **G**) Inverse analysis to F to identify motif enrichment of motifs that are not found in CNEs that show accessibility in IECs, suggesting these transcription factors and motifs are less likely to play a specific role in conserved IEC biology.
(PDF)

S9 Fig Remarkable IEC-specific accessible chromatin of a conserved enhancer region upstream of HES1 in multiple species. **A**) WashU Epigenome browser screen shot of HES1 locus for a large number of accessible chromatin datasets from diverse tissues for the Human roadmap study and human colon IECs from this study shows exclusive accessibility for hzCNE_44665 in intestinal datasets. **B**) WashU Epigenome browser screen shot of Hes1 locus for a large number of accessible chromatin datasets from diverse tissues for the mouse ENCODE study and mouse ileum and colon IECs used for this study shows exclusive accessibility for mzCNE_44665 in intestinal datasets. **C**) Schematic for common motifs found in CNE_44665 shows a common placement and order of HNF1, RBPJ, and HIF2B TFBS across zebrafish, mouse and human overlayed on the CNE region (bronze) and the neighboring region (light gray). Solid lines between predicted TFBS are drawn to represent the presumed conservation of these sites. Interestingly, a putative GFI1B binding site detected in zCNE_44665 is absent in mzCNE_44665 and hzCNE_44665, but a GFI1B site can be found in the neighboring region that still shows IEC accessible chromatin specificity in human and mouse (see A and B). This relationship is marked with a dashed line.
(PDF)

S10 Fig Additional characterization of regulatory capacity of hes1 zCNE_44665 in transgenic zebrafish. **A**) 7 dpf cross-section of individual transgenic zebrafish lines for hes1 Tg(zCNE_44665:GFP) show consistent GFP expression in a subset of IECs often at the base of nascent folds. **B**) Minimal gross overlap can be seen between Notch positive cells in the pancreas and the Tg(zCNE_44665:GFP) signal in 7 dpf zebrafish **C**) The corpusles of Stannius show high Notch positive signaling and little to no GFP expression from Tg(zCNE_44665:GFP). **D**) Overlap can be seen between Notch positive cells and Tg(zCNE_44665:GFP) signal in the liver. **E**) and **F**) Examples of more apical nuclei in 8 week old cross-section in Tg(zCNE_44665:GFP) and Notch positive cells. **G**) Whole-mount stereoscopic images of 7 dpf Tg(zCNE_44665:GFP)/Tg(-4.5fabp2:DsRed) shows a large proportion of IECs are DsRed+ enterocytes, however a subset of the IECs not expressing DsRed are GFP+ and are marked by white arrows. **H**) Whole-mount stereoscopic images of 7 dpf Tg(zCNE_44665:GFP)/Tg(neurod1:TagRFP) show lack of overlap between the hes1 Tg(zCNE_44665:GFP) and enteroendocrine cells. Representative GFP+ cells are marked by a yellow arrow and RFP+ cells are marked by a red arrow. Pancreatic islet marked by i.
(PDF)

S1 Table Table of 1-to-1-to-1-to-1 orthologs included in identification of IEC signature genes. Table of 1-to-1-to-1-to-1 orthologs included in identification of IEC signature genes including PC1 correlation used as a cutoff for IEC signature gene inclusion. Included are FPKM values for other tissues [29] and for 3 replicates of IECs. Also included are disease terms associated with IEC signature genes from DIOPT (http://www.flyrnai.org/diopt).
(XLSX)

S2 Table Summary table of DAVID and Enrichr analysis using IEC signature genes as input and remaining 1-to-1-to-1-to-1 orthologs used in identifying IEC signatures from S1 Table as background. (XLSX)

S3 Table Summary table of peaks identified and data sets used in this study. (XLSX)

S4 Table Primer sequences and coordinates in this study. (XLSX)

S5 Table Zebrafish transgenic lines generated for this study. (XLSX)

S1 Movie Confocal cross-section z-stack movie shows overlap between hes1 Tg(zCNE_44665:GFP) and Notch signaling Tg(EPV.Tp1-Ocu.Hbb2:hmgb1-mCherry). (AVI)

## Abbreviations

*Ada*: adenosine deaminase
AT%: the percentage of DNA bases that are either adenosine or thymine
CArG: CA/T-rich-G
*CDH17*: cadherin 17
CNE: conserved nonexonic elements
DNase-seq: DNase I hypersensitive sites sequencing
DsRed: Discosoma sp. red fluorescent protein
ENCODE: Encyclopedia of DNA Elements
ETS: E26 transformation specific
*FABP6*: fatty acid binding protein 6
FAIRE-seq: Formaldehyde-Assisted Isolation of Regulatory Elements sequencing
FPKM: Fragments Per Kilobase of transcript per Million mapped reads
*GALNT6*: polypeptide N-acetylgalactosaminyltransferase 6
GO: gene ontology
*her6*: hairy-related 6
*HES1*: hairy and enhancer of split-1
*HNF1*: hepatocyte nuclear factor 1
IBD: Inflammatory Bowel Disease
IEC: intestinal epithelial cell
MADS: MCM1, AGAMOUS, DEFICIENS, and SRF
MAF: multiple alignment format
PC: principle component
PCA: principal component analysis
PWM: position weight matrices
*PYY*: peptide YY
*RBP2*: retinol binding protein 2
RBPJ: recombination signal binding protein for immunoglobulin kappa J region
RNA-seq: RNA sequencing
TF: transcription factor
TFBS: transcription factor binding sites
TSS: transcription start site
TTS: transcription termination site
UCSC: University of California Santa Cruz
WT: wild-type
zCNE: zebrafish conserved nonexonic elements
