# Supplementary figures and images for: Genomic dissection of conserved transcriptional regulation in intestinal epithelial cells
Source: PLoS Biol. 2017 Aug 29;15(8):e2002054. doi: 10.1371/journal.pbio.2002054 (PMC5574553; doi:10.1371/journal.pbio.2002054)

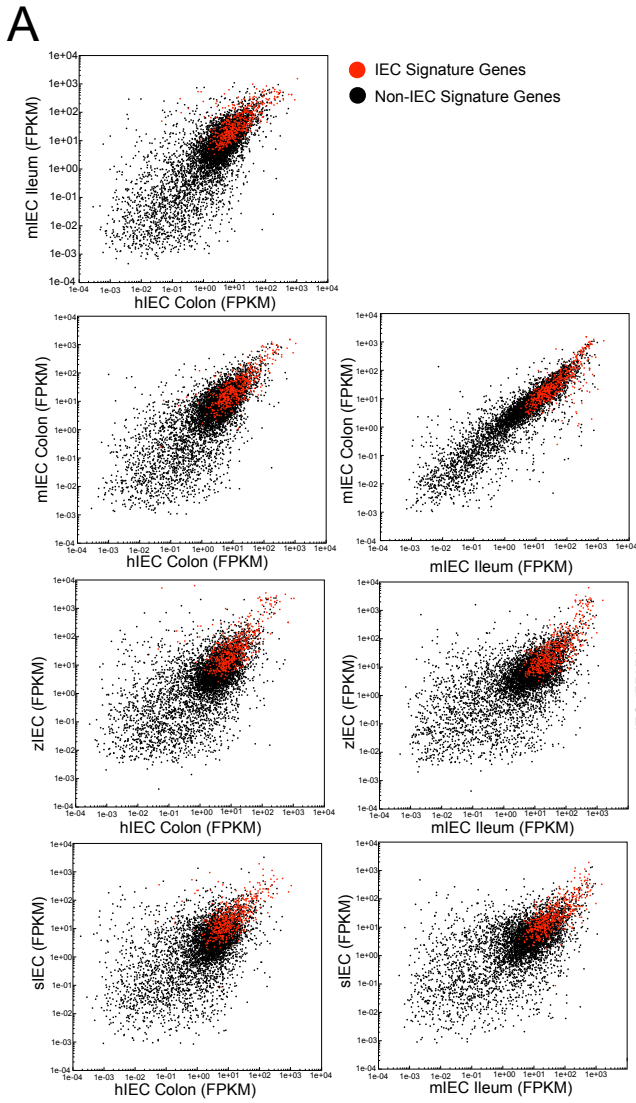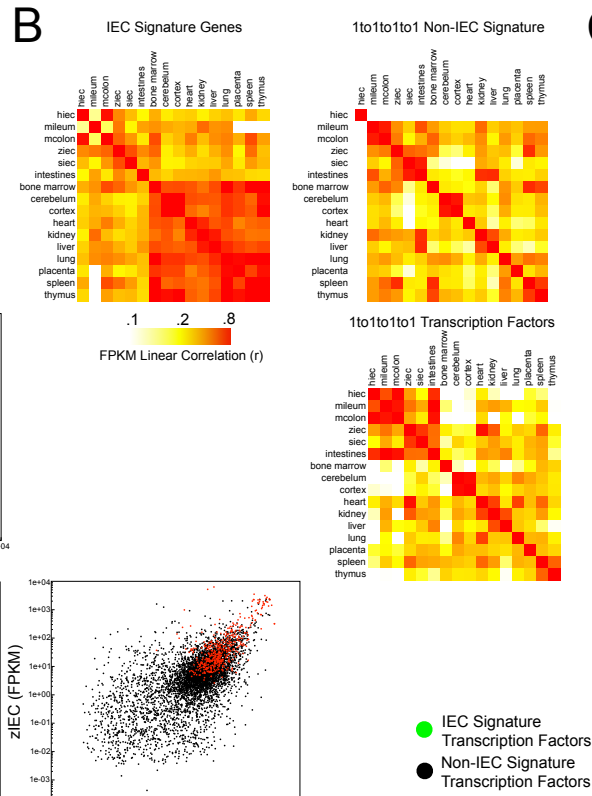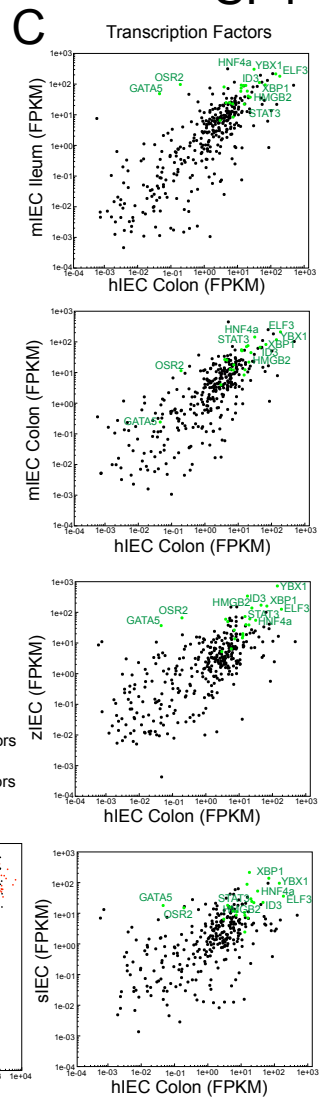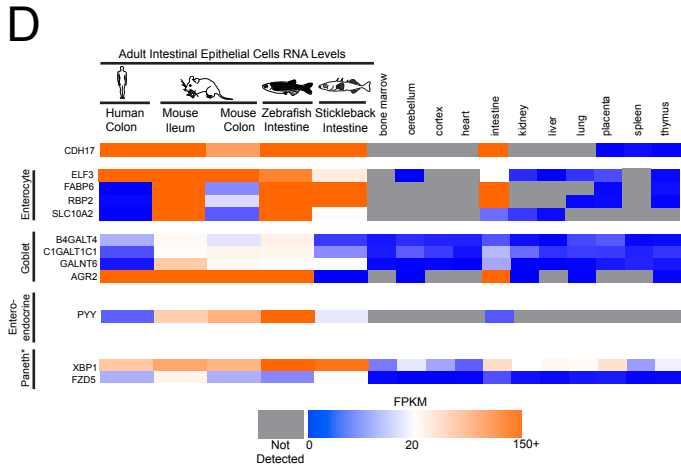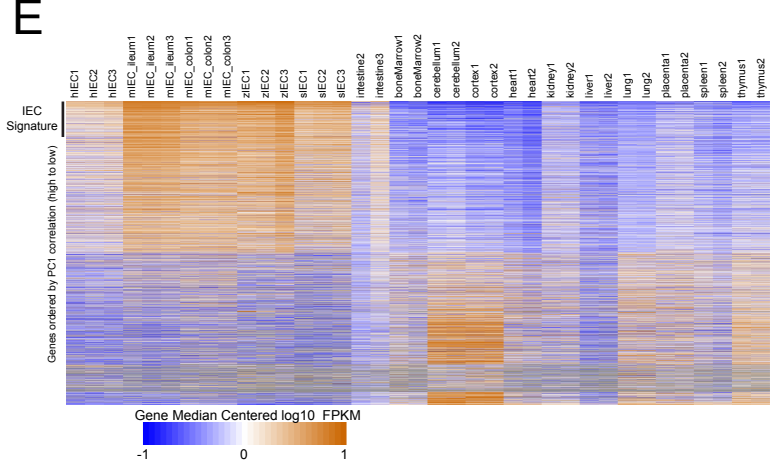

Supplement: S1 Fig — A) Pairwise scatter plots showing FPKM values for IEC datasets from four species for IEC signature genes (red) and non-IEC signature genes (black). B) Heatmap of linear correlation coefficients for pairwise comparison between IEC datasets and mouse non-IEC datasets [29] for IEC signature genes, non-IEC signature genes, and transcription factors. C) Scatter plots of IEC FPKM values for IEC signature (green) and non-IEC signature (black) transcription factors. D) Heatmap of FPKM values or IEC and other tissues for representative genes that are specific to IEC subtypes. Despite the lack of Paneth cells in zebrafish, XBP1, a Paneth cell associated transcript, is highly expressed in zebrafish IECs. E) Heat map of expression levels for IECs and other non-IEC tissues [29] ordered by PC1 correlations from Fig 1E. Black vertical bar marks IEC signature genes. (PDF) [file pbio.2002054.s001.pdf]

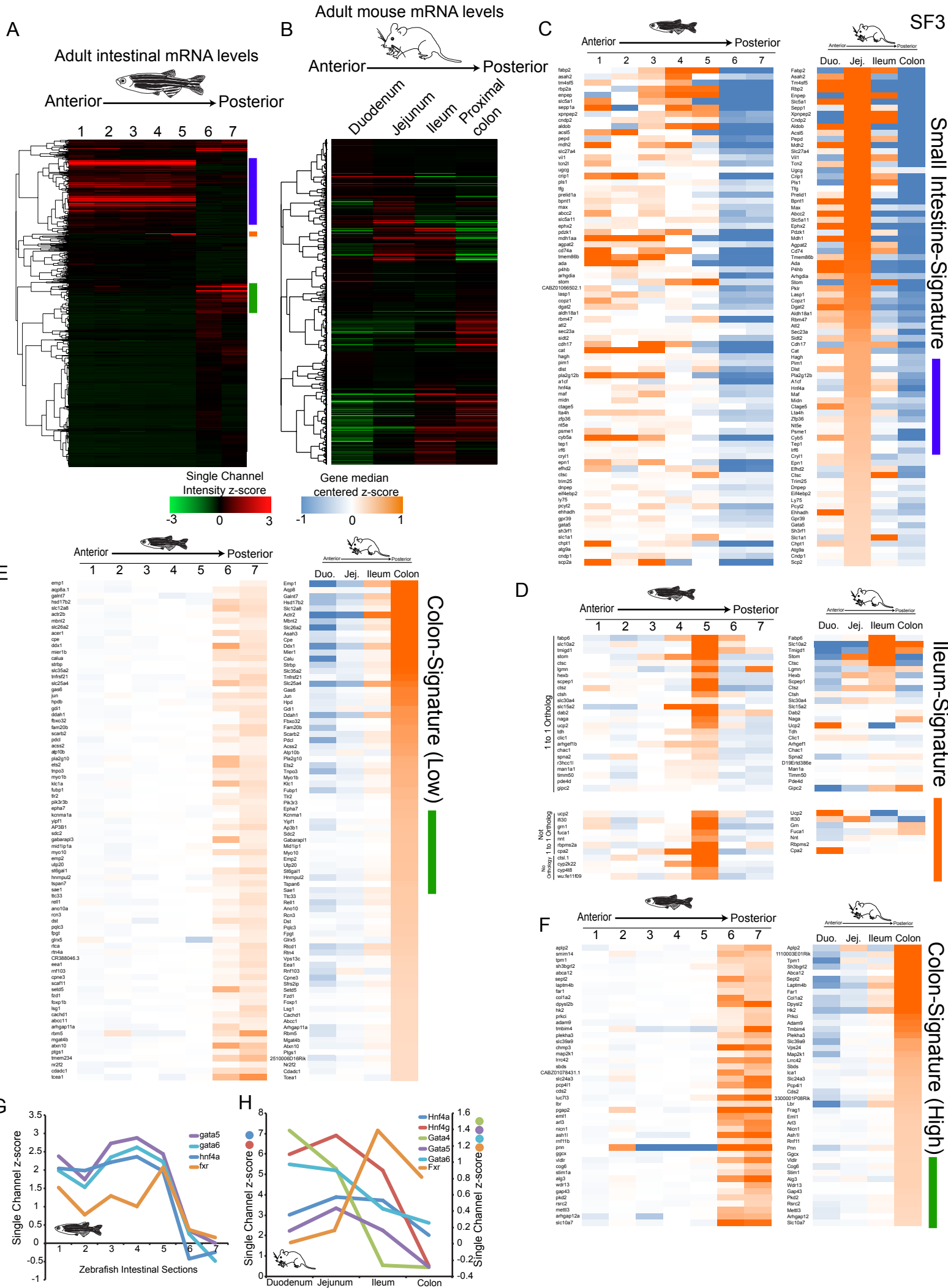

Supplement: S3 Fig — A) Consistent with a previously published result [15], a heatmap of a cluster analysis of previously published single channel intensity z-scored mircroarray mRNA levels from adult zebrafish whole intestine dissected into 7 equal length segments. Cluster analysis includes zebrafish 1to1 orthologs (zebrafish to mouse) with at least one intestine section’s z-score value greater than 0 clustered using a complete clustering method with an uncentered similarity metric in Cluster 3.0. B) Same as A for adult mouse whole intestine sections from using previously published mouse data [47]. C) Heatmap of 1to1 orthologs from the zebrafish cluster in (A) marked by a blue bar for genes with a linear correlation over 0.6 between the 7 zebrafish sections and 7 values generated by linear interpolation between the z-scores for the 4 mouse segments sorted by z-score in mouse jejunum. Evidence for conserved transcriptional regulation for genes most highly expressed in zebrafish sections 4–5 and mouse jejunum suggest nuanced expression patterns are conserved. D) Same as C for the orange cluster highlighting an apparent ileum signature. Additional zebrafish genes of potential interest due to their expression patterns, but without 1to1 orthology, are added by hand. E-F) Same as (C) for the green cluster broken into two groups of genes that are lowly (E) or highly (F) relatively expressed preferentially in zebrafish sections 6–7 sorted by values from the mouse colon. Single channel z-score values across intestinal segments in (G) zebrafish and (H) mouse for transcription factors that have binding sites in the promoters of Rbp2 and Fabp6 show expression patterns that may help specify regional intestinal expression patterns in IECs across species. Colored dots on y-axes correspond to scales for colored data sets. Numerical values can be found in S1 Table. (PDF) [file pbio.2002054.s003.pdf]

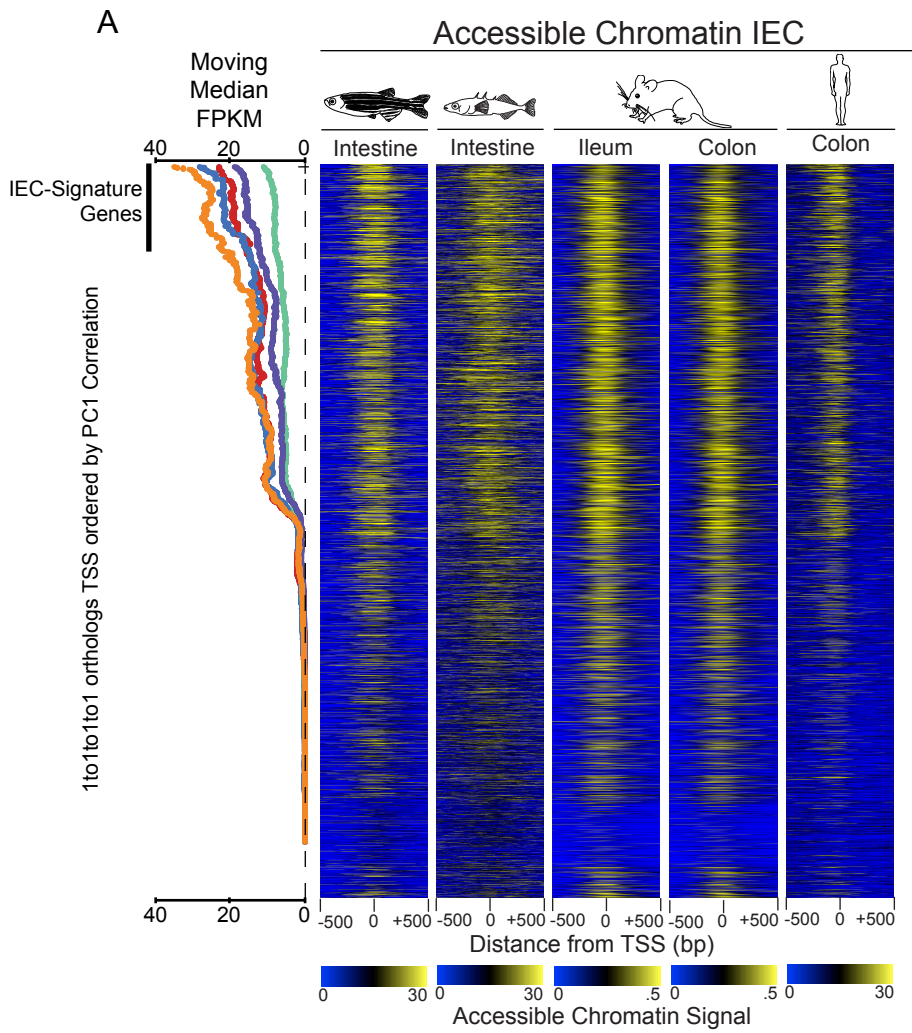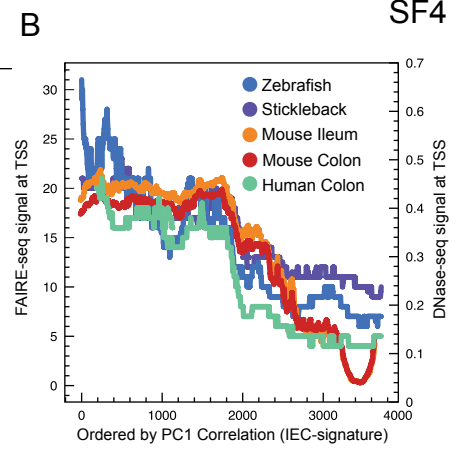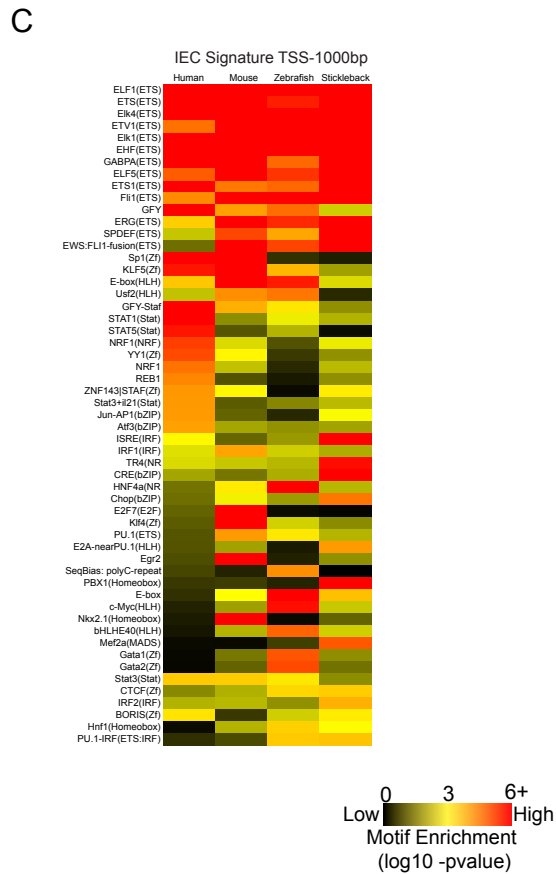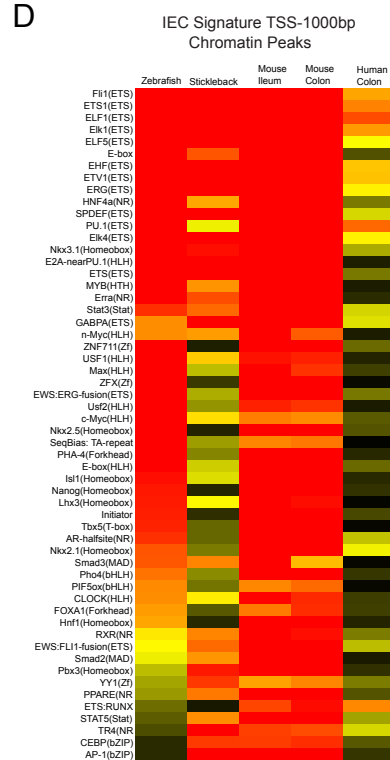

Supplement: S4 Fig — A) Accessible chromatin signal at 1000 bp surrounding the TSS of 1to1to1to1 orthologs ordered by PC1 correlation used to identify IEC signature genes for zebrafish, stickleback, mouse ileum, mouse colon and human colon accessible chromatin data (Right). Moving median (Left) for FPKM of associated genes (250 gene window, 1 step; color scheme used throughout and shown in B) IEC signature genes are marked by a black vertical bar. B) Moving median (250 gene window, 1 step) for accessible chromatin signal at TSS from IECs based on ordering in A). Numerical values can be found in S1 Table. C) Heatmap of common motif enrichment within the TSS-1000 bp region for IEC signature genes. D) Heatmap of common motif enrichment within IEC accessible chromatin peaks within the region TSS-1000 bp for IEC signature genes. (PDF) [file pbio.2002054.s004.pdf]

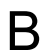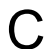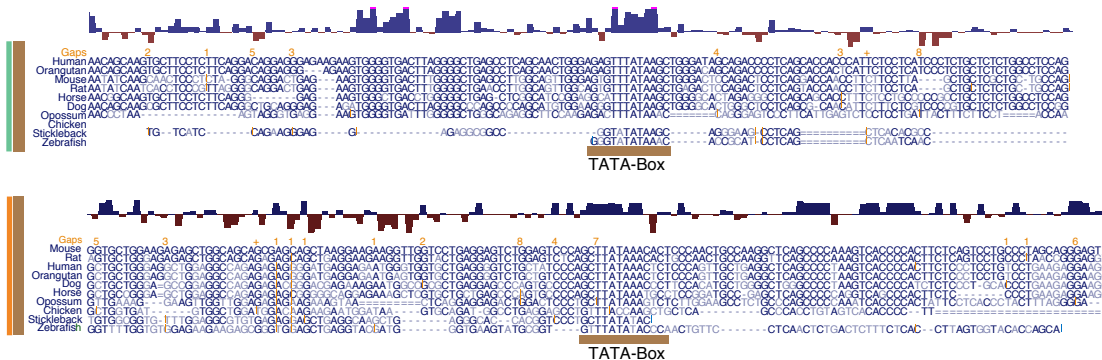

Supplement: S5 Fig — A) Accessible chromatin signal for RBP2(a) human, mouse, zebrafish and stickleback loci. Accessible chromatin peaks colored based on color scheme used throughout for IEC samples, conservation from zebrafish to human or mouse; bronze, and cloned region; black). B) Accessible chromatin signal for FABP6 loci from human, mouse, zebrafish and stickleback. Accessible chromatin peaks colored based on color scheme used throughout for IEC samples, conservation from zebrafish to human or mouse; bronze, and cloned regions; black). C) UCSC screenshot for Phylop conservation score and Multiz hg19 alignment (top) and Phylop conservation score for Multiz mm9 alignment (bottom) for representative species at the region immediately upstream of FABP6/Fabp6 transcription start site and highlighted in bronze in B. Highlighted are predicted TATA-box motifs that overlap the conserved region in both mouse and human. While a conserved signal is detected from human and mouse to zebrafish only the TATA-box appears to be conserved. (PDF) [file pbio.2002054.s005.pdf]

A

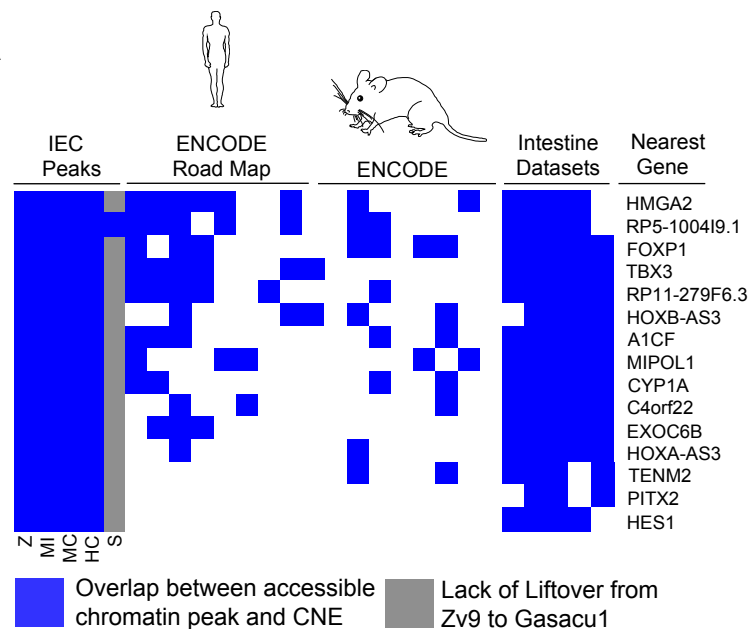

B

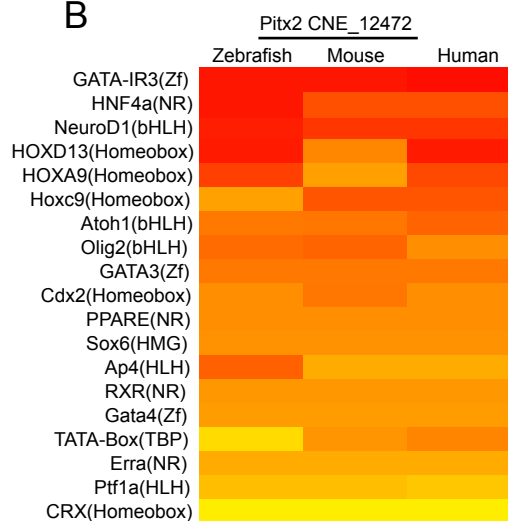

C

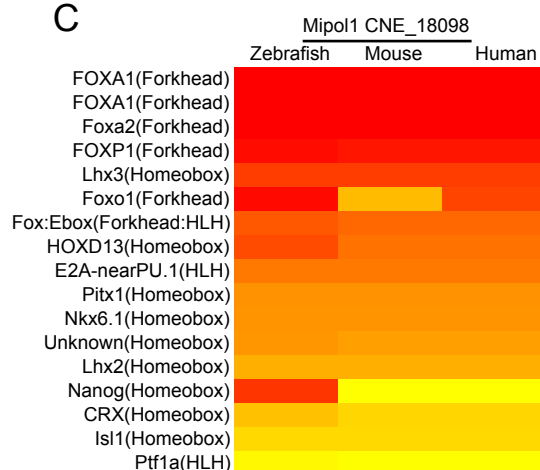

D

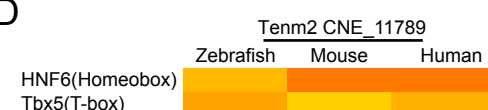

E

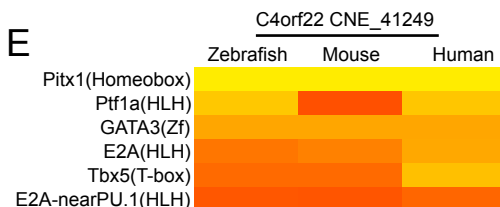

F

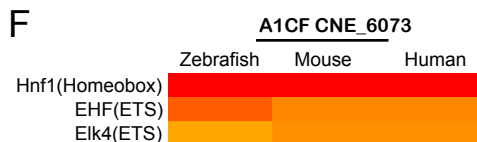

G

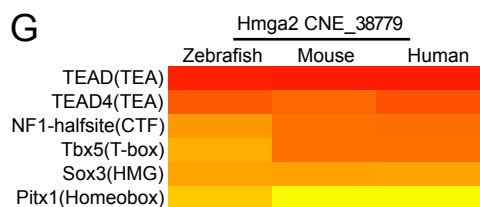

H

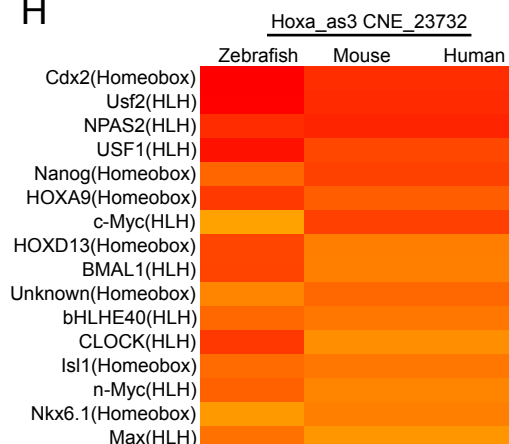

I

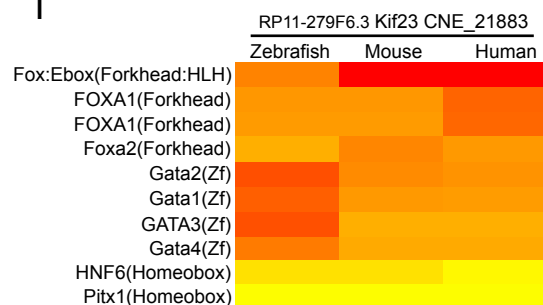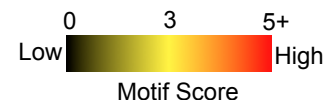

J

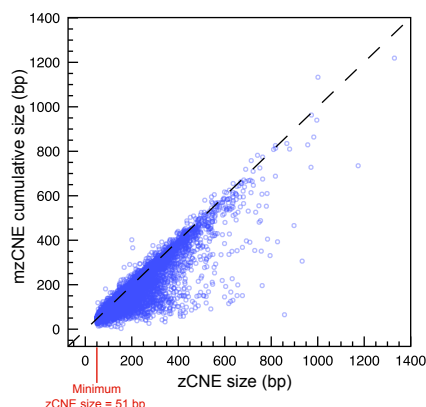

K

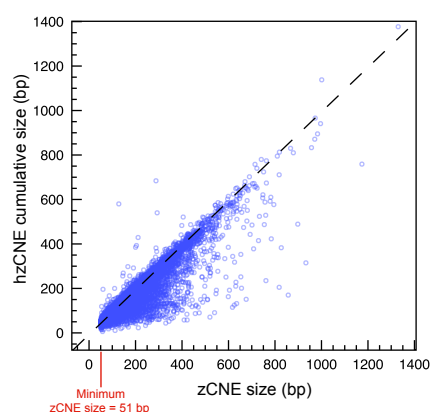

L

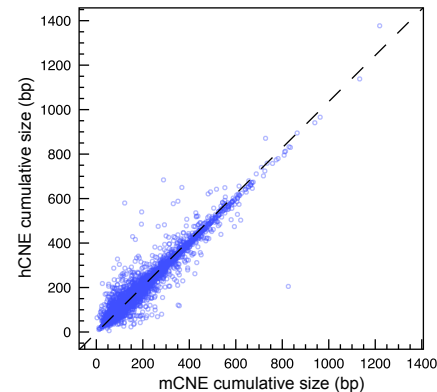

Supplement: S7 Fig — A) CNEs showing accessible chromatin largely in IECs corresponding to Fig 6C and 6E. Common results of Homer transcription factor binding site motif search in all three species for CNEs B) PITX2 CNE_12472, C) MIPOL1 CNE_18098, D) TENM2 CNE_11789, E) C4ORF22 CNE_41249, F) A1CF CNE_6073, G) HMGA2 CNE_38779, H) HOX3_AS3 CNE_23732, and I) RP11-279F6.3/KIF23 CNE_21883. Not pictured are CNEs that had no motifs in common in all three species. Corresponding CNE size [55] between J) zebrafish and mouse, K) zebrafish and human, and L) mouse and humans reveals that linked CNE size is often smaller when comparing from the zebrafish anchor genome to alignment genomes suggesting conserved regulatory information may be lost using conservation strategies. Dashed black line represents a slope of 1. In a few cases CNEs from zebrafish can map to non-continuous regions of the alignment genomes. In this case, the cumulative size of these regions is summed to generate a single size for each corresponding CNE in each genome. (PDF) [file pbio.2002054.s007.pdf]

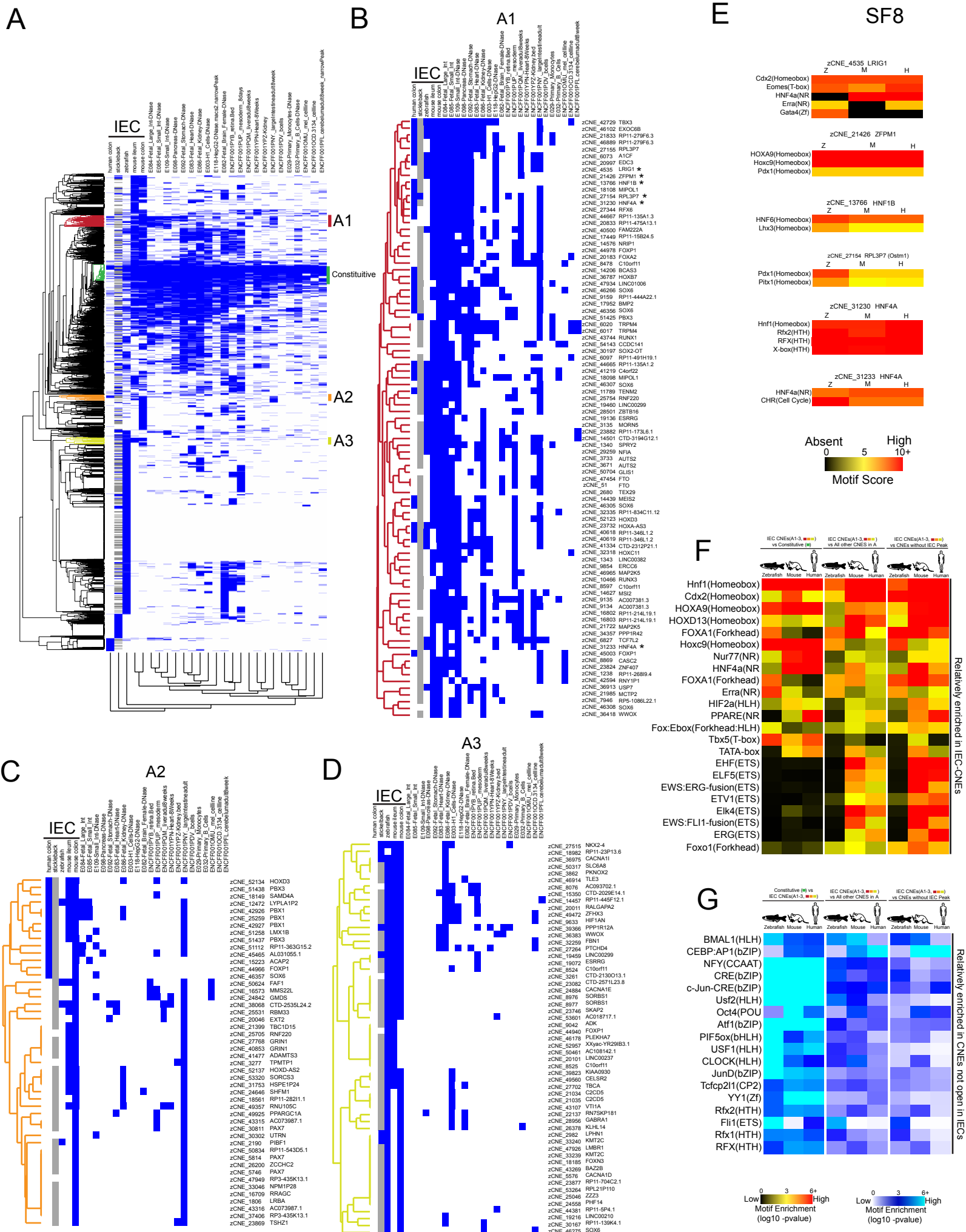

Supplement: S8 Fig — A) Heatmap of cluster analysis of overlaps between CNE regions and accessible chromatin peak calls for IECs and other tissues. Clusters showing CNEs with relatively specific accessible chromatin in IECs are marked as A1 (red), A2 (orange), and A3 (yellow). CNEs that appear largely constitutively accessible in IEC and other tissues in all species are labeled Constitutive (green). B) Blow up of A1 cluster showing CNE names [55] and nearest genes. CNEs marked with asterisks are used in motif analysis in E. C) Same as B for A2 cluster. D) Same as B for A3 cluster. E) Common motifs found in CNEs identified in A-D for a subset of CNEs near genes of interest based on known IEC biology or due to the specificity of accessible chromatin to IECs in multiple species. F) Motif enrichment to identify motifs that are more often found in CNEs that show chromatin accessibility in IECs reveals multiple TFBSs important in IEC biology like HNF1, CDX2, and HNF4A. Various groups of non-IEC specific CNEs are used as backgrounds for motif enrichment as labeled. G) Inverse analysis to F to identify motif enrichment of motifs that are not found in CNEs that show accessibility in IECs, suggesting these transcription factors and motifs are less likely to play a specific role in conserved IEC biology. (PDF) [file pbio.2002054.s008.pdf]

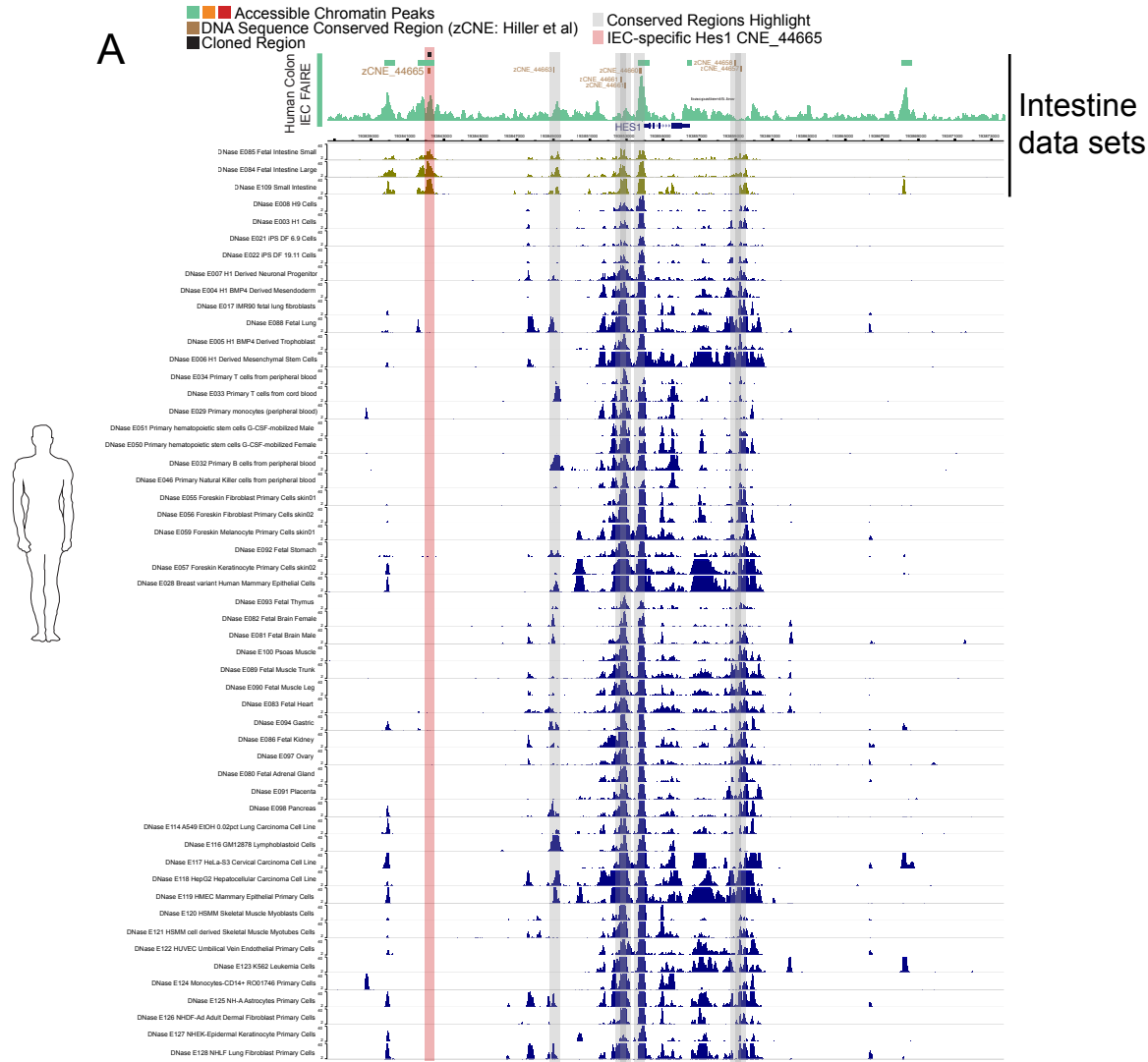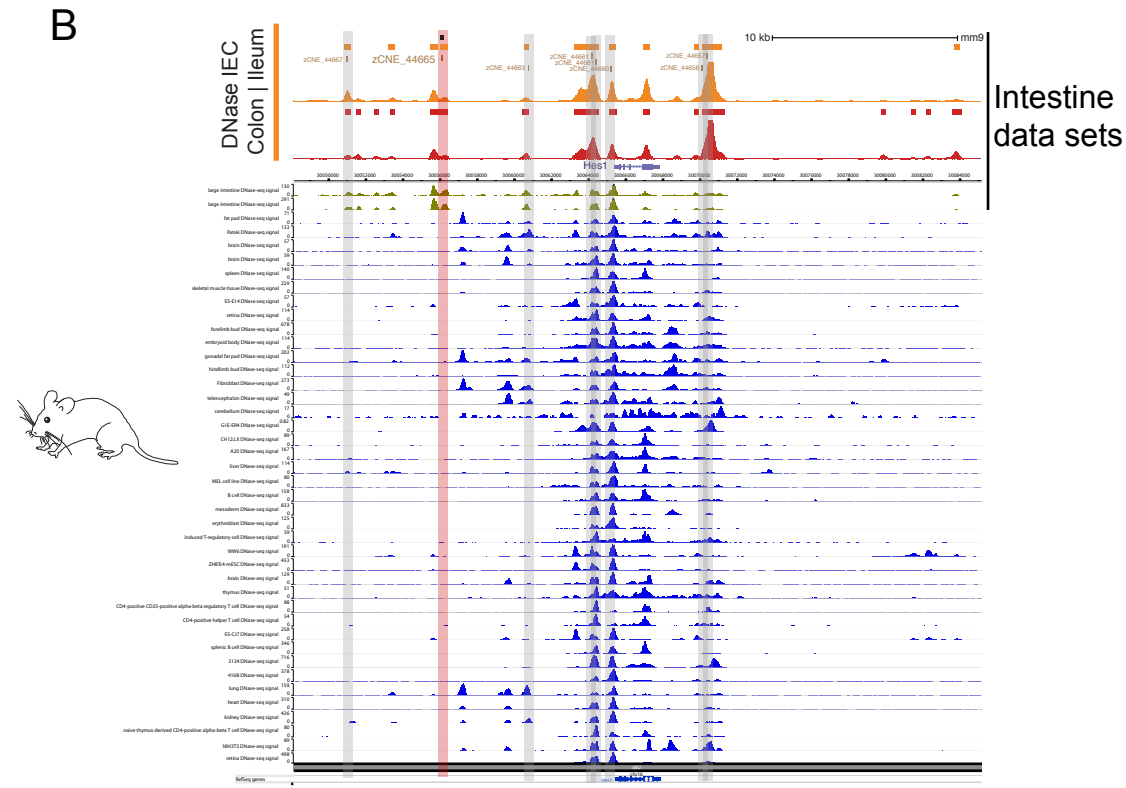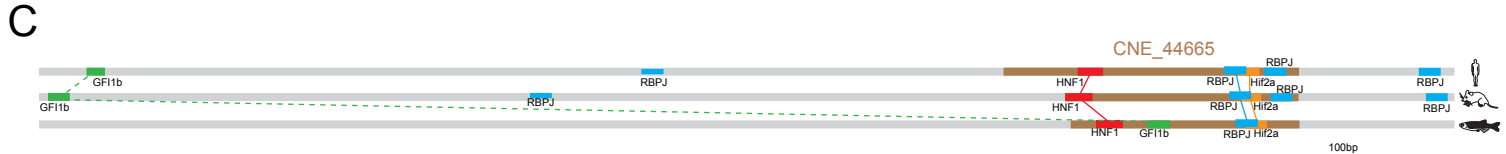

Supplement: S9 Fig — A) WashU Epigenome browser screen shot of HES1 locus for a large number of accessible chromatin datasets from diverse tissues for the Human roadmap study and human colon IECs from this study shows exclusive accessibility for hzCNE_44665 in intestinal datasets. B) WashU Epigenome browser screen shot of Hes1 locus for a large number of accessible chromatin datasets from diverse tissues for the mouse ENCODE study and mouse ileum and colon IECs used for this study shows exclusive accessibility for mzCNE_44665 in intestinal datasets. C) Schematic for common motifs found in CNE_44665 shows a common placement and order of HNF1, RBPJ, and HIF2B TFBS across zebrafish, mouse and human overlayed on the CNE region (bronze) and the neighboring region (light gray). Solid lines between predicted TFBS are drawn to represent the presumed conservation of these sites. Interestingly, a putative GFI1B binding site detected in zCNE_44665 is absent in mzCNE_44665 and hzCNE_44665, but a GFI1B site can be found in the neighboring region that still shows IEC accessible chromatin specificity in human and mouse (see A and B). This relationship is marked with a dashed line. (PDF) [file pbio.2002054.s009.pdf]

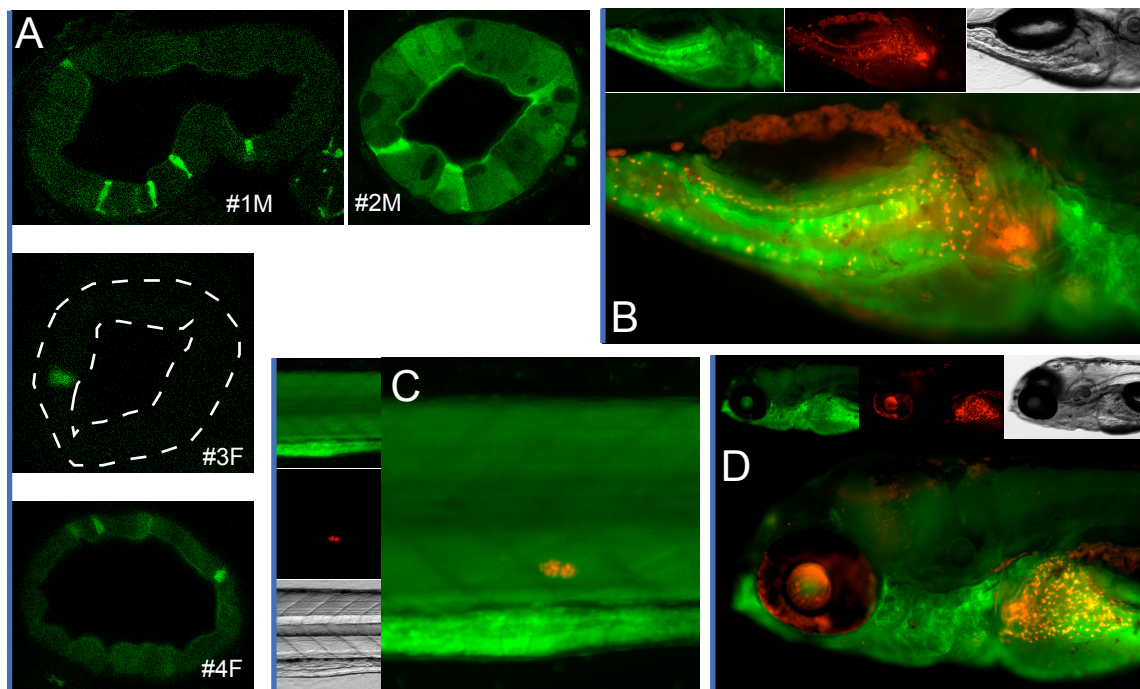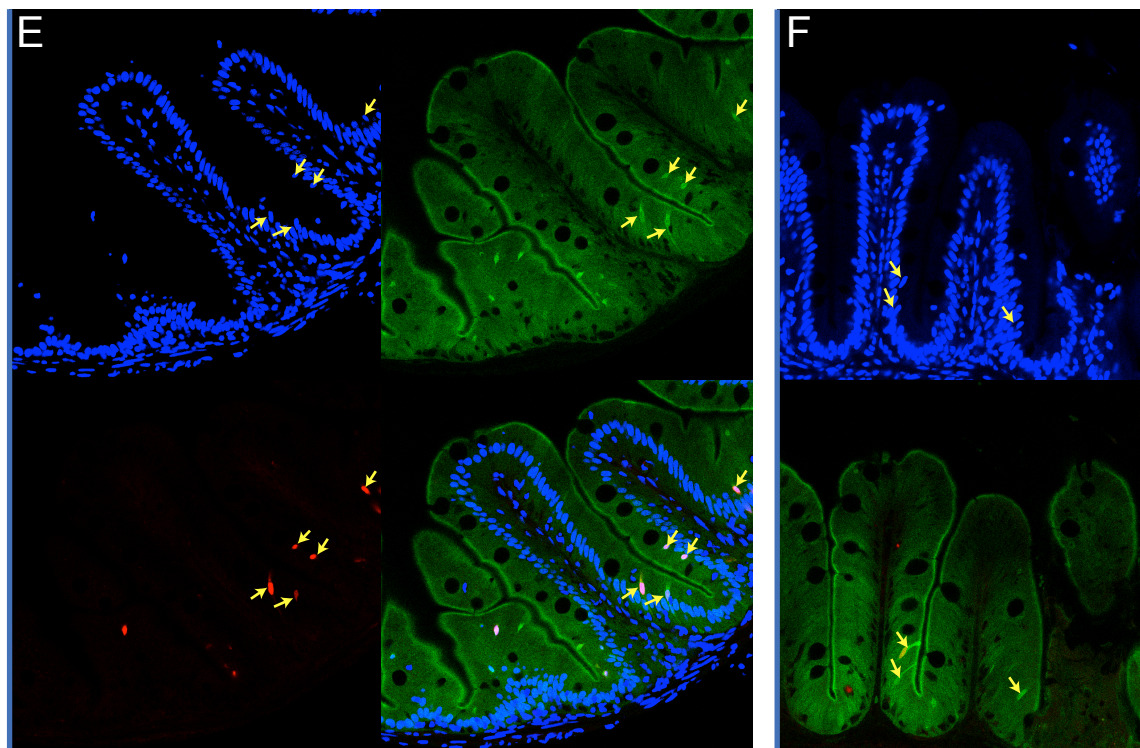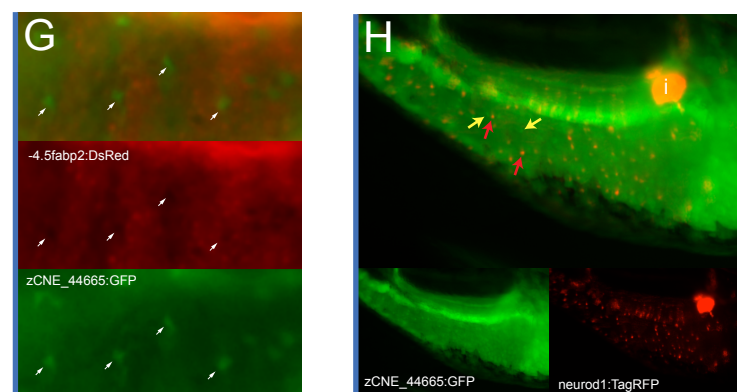

Supplement: S10 Fig — A) 7 dpf cross-section of individual transgenic zebrafish lines for hes1 Tg(zCNE_44665:GFP) show consistent GFP expression in a subset of IECs often at the base of nascent folds. B) Minimal gross overlap can be seen between Notch positive cells in the pancreas and the Tg(zCNE_44665:GFP) signal in 7 dpf zebrafish C) The corpusles of Stannius show high Notch positive signaling and little to no GFP expression from Tg(zCNE_44665:GFP). D) Overlap can be seen between Notch positive cells and Tg(zCNE_44665:GFP) signal in the liver. E) and F) Examples of more apical nuclei in 8 week old cross-section in Tg(zCNE_44665:GFP) and Notch positive cells. G) Whole-mount stereoscopic images of 7 dpf Tg(zCNE_44665:GFP)/Tg(-4.5fabp2:DsRed) shows a large proportion of IECs are DsRed+ enterocytes, however a subset of the IECs not expressing DsRed are GFP+ and are marked by white arrows. H) Whole-mount stereoscopic images of 7 dpf Tg(zCNE_44665:GFP)/Tg(neurod1:TagRFP) show lack of overlap between the hes1 Tg(zCNE_44665:GFP) and enteroendocrine cells. Representative GFP+ cells are marked by a yellow arrow and RFP+ cells are marked by a red arrow. Pancreatic islet marked by i. (PDF) [file pbio.2002054.s010.pdf]
