# Supplementary material for: Genomic dissection of conserved transcriptional regulation in intestinal epithelial cells
Source: PLoS Biol. 2017 Aug 29;15(8):e2002054. doi: 10.1371/journal.pbio.2002054 (PMC5574553; doi:10.1371/journal.pbio.2002054)
Supplement: S2 Fig — A) Heatmap of clustergram using complete linkage with a correlation uncentered similarity metric for arrays using log10 FPKM values from IEC datasets for all species for 1to1to1to1 orthologs. Genes are ordered by average log10 FPKM values across all replicates. B) Heatmap of clustergram using complete linkage with a correlation uncentered similarity metric for arrays and genes using log10 FPKM values for all genes from stickleback IECs. C) Same as B for zebrafish IECs D) Same as B for human IECs. E) Heatmap of clustergram using complete linkage with a correlation uncentered similarity metric for arrays and genes using the sum of accessible chromatin signal (sequencing counts) at the TSS+/-50 bp (the 100 bp window centered on the TSS) for 1-1-1-1 orthologs. Arrays are median centered and normalized using Cluster 3.0. F) The same as E for zebrafish IECs. Stickleback data is also provided and ordered by the zebrafish clustering. G) The same as E for human IECs. (PDF) [file pbio.2002054.s002.pdf]

SF2

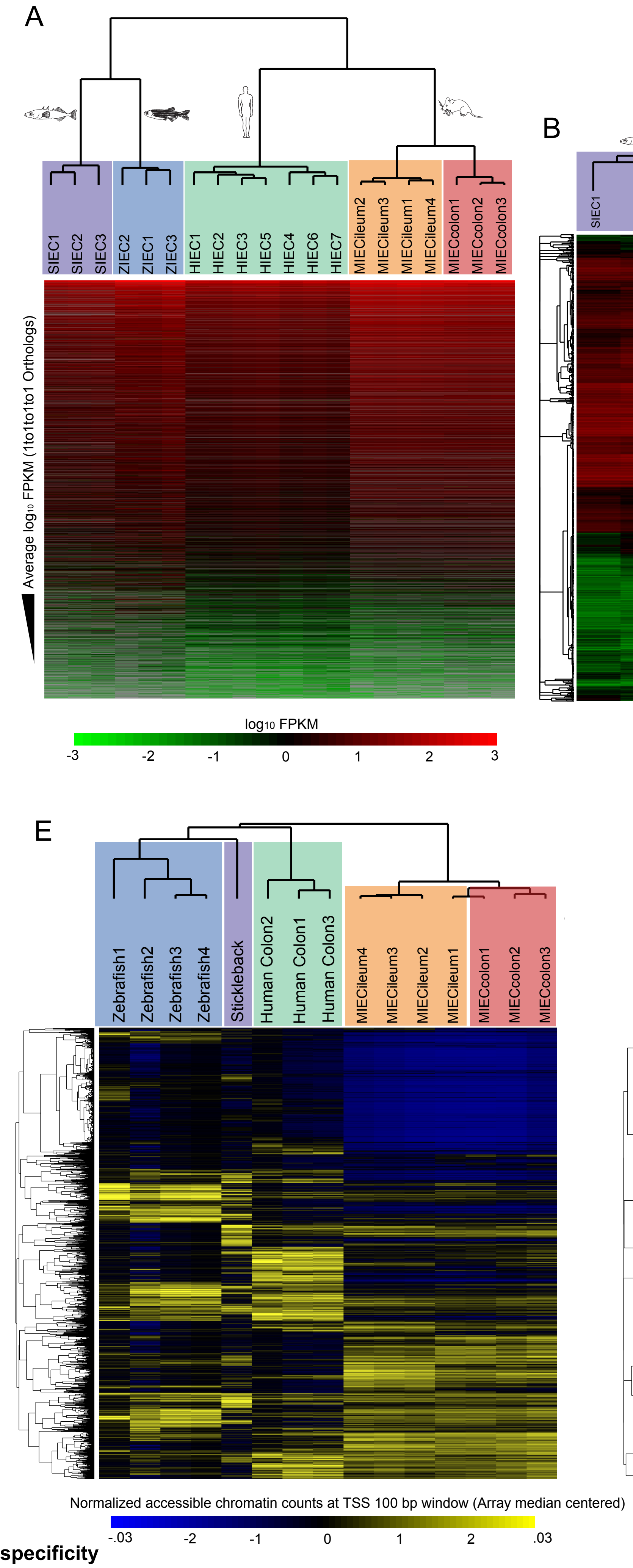
A
SIEC1
SIEC2
SIEC3
ZIEC2
ZIEC1
ZIEC3
HIEC1
HIEC2
HIEC3
HIEC5
HIEC4
HIEC6
HIEC7
MIECileum2
MIECileum3
MIECileum1
MIECileum4
MIECcolon1
MIECcolon2
MIECcolon3
Average log10 FPKM (1to1to1to1 Orthologs)
log10 FPKM
-3
-2
-1
0
1
2
3
B
SIEC1
SIEC2
SIEC3
C
ZIEC1
ZIEC2
ZIEC3
D
HIEC1
HIEC2
HIEC4
HIEC3
HIEC5
HIEC6
HIEC7
E
Zebrafish1
Zebrafish2
Zebrafish3
Zebrafish4
Stickleback
Human Colon2
Human Colon1
Human Colon3
MIECileum4
MIECileum3
MIECileum2
MIECileum1
MIECcolon1
MIECcolon2
MIECcolon3
F
Zebrafish1
Zebrafish2
Zebrafish3
Zebrafish4
Stickleback1
G
Human Colon2
Human Colon1
Human Colon3
Normalized accessible chromatin counts at TSS 100 bp window (Array median centered)
-.03
-2
-1
0
1
2
.03
specificity
