# Supplementary material for: Genomic dissection of conserved transcriptional regulation in intestinal epithelial cells
Source: PLoS Biol. 2017 Aug 29;15(8):e2002054. doi: 10.1371/journal.pbio.2002054 (PMC5574553; doi:10.1371/journal.pbio.2002054)
Supplement: S6 Fig — A) Simplified schematic of 4-way LR recombination cloning. Briefly, fragments amplified from a genomic template with primers containing FseI and AscI overhangs are cloned into a 5’ entry vector. A 4-way LR reaction combines the 5’ entry containing the putative regulatory element with a middle entry vector containing the minimal cFos promoter (S4 Table) and 3’ entry vector with eGFP into a destination vector containing Tol2 insertion sites [125]. B) A cFos control vector was generated to test for potential insertion background expression and the expression capacity of the cFos minimal promoter in IECs as this construct presumably can act as an enhancer trap when inserted into the genome without additional regulatory information. Comprehensive testing of potential insertion influence with this control vector is difficult, however typically no or little expression in IECs was found and none of the cFos control expression fish drove expression patterns that were consistent with the elements tested in the main body of the paper (C-F). While wholemount stereoscopic microscopy of fish containing this control construct did not readily detect IEC GFP expression that we found when testing putative IEC regulatory elements (Figs 4–6). We also performed confocal microscopy on a subset of fish as this is typically more sensitive at identifying localized GFP signal. We note below where limited IEC expression existed in the control lines we profiled, however any IEC expression was not usual and not consistent between control lines. C) Developmental pattern of GFP expression of cFos control line 3m. D) Developmental pattern of GFP expression of cFos control F1 line 7m D’) Cross-section of cFos control line 7m shows light expression in a goblet cell. E) Developmental pattern of GFP expression of cFos control F1 line 9m. E’) Cross-section of cFos control line 9m shows no detectable GFP expression in IECs. F) Developmental pattern of GFP expression of cFos control F1 line 9m. F’) Cross [file pbio.2002054.s006.pdf]

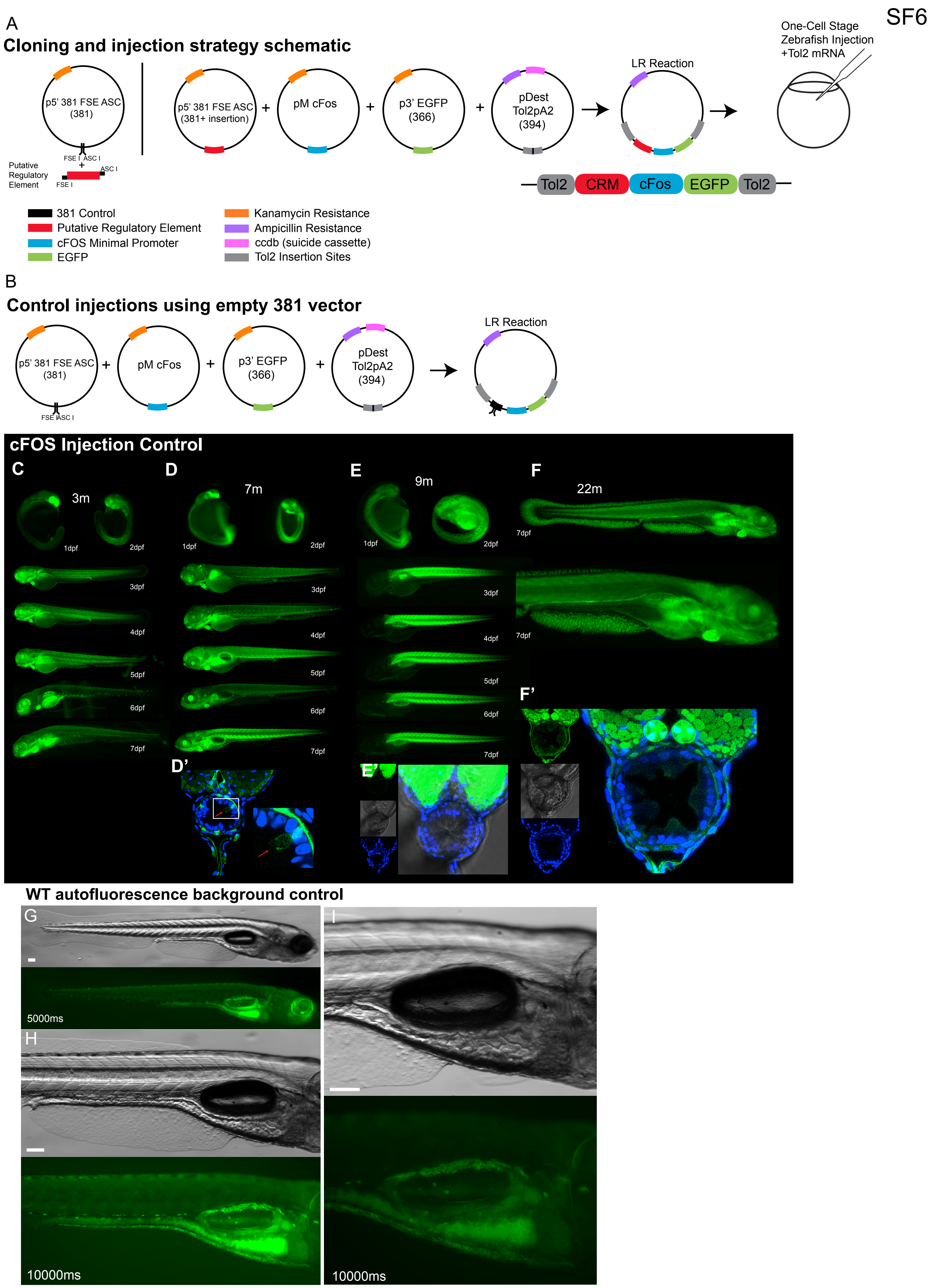
SF6
A
Cloning and injection strategy schematic
p5' 381 FSE ASC (381)
FSE I ASC I
Putative Regulatory Element
FSE I
ASC I
p5' 381 FSE ASC (381+ insertion)
pM cFos
p3' EGFP (366)
pDest Tol2pA2 (394)
LR Reaction
One-Cell Stage Zebrafish Injection +Tol2 mRNA
Tol2
CRM
cFos
EGFP
Tol2
381 Control
Putative Regulatory Element
cFOS Minimal Promoter
EGFP
Kanamycin Resistance
Ampicillin Resistance
ccdb (suicide cassette)
Tol2 Insertion Sites
B
Control injections using empty 381 vector
p5' 381 FSE ASC (381)
pM cFos
p3' EGFP (366)
pDest Tol2pA2 (394)
LR Reaction
FSE I ASC I
cFOS Injection Control
C
3m
1dpf
2dpf
3dpf
4dpf
5dpf
6dpf
7dpf
D
7m
1dpf
2dpf
3dpf
4dpf
5dpf
6dpf
7dpf
D'
E
9m
1dpf
2dpf
3dpf
4dpf
5dpf
6dpf
7dpf
E'
F
22m
7dpf
7dpf
F'
WT autofluorescence background control
G
5000ms
H
10000ms
I
10000ms
